# Supplementary figures and images for: Clinical manifestations of Rift Valley fever in humans: Systematic review and meta-analysis
Source: PLoS Negl Trop Dis. 2022 Mar 25;16(3):e0010233. doi: 10.1371/journal.pntd.0010233 (PMC8986116; doi:10.1371/journal.pntd.0010233)

**S1 Fig. RVF prevalence among included studies that reported patients by gender**

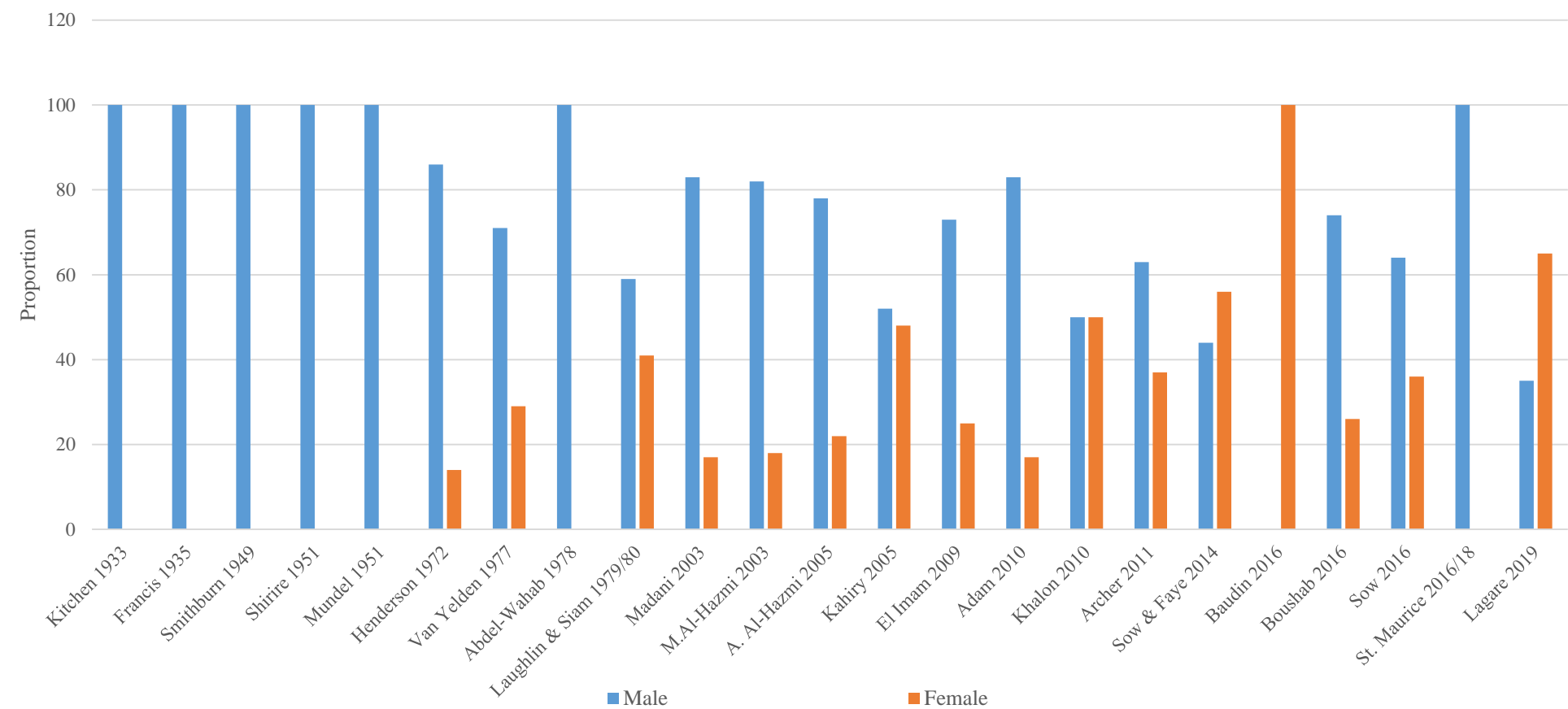

Supplement: S1 Fig — [5,8,9,17,21,22,31–37,39–43,45,47–50,53,55]. (PDF) [file pntd.0010233.s001.pdf]

**S2 Fig. Distribution of RVF outbreaks in a year for included studies**

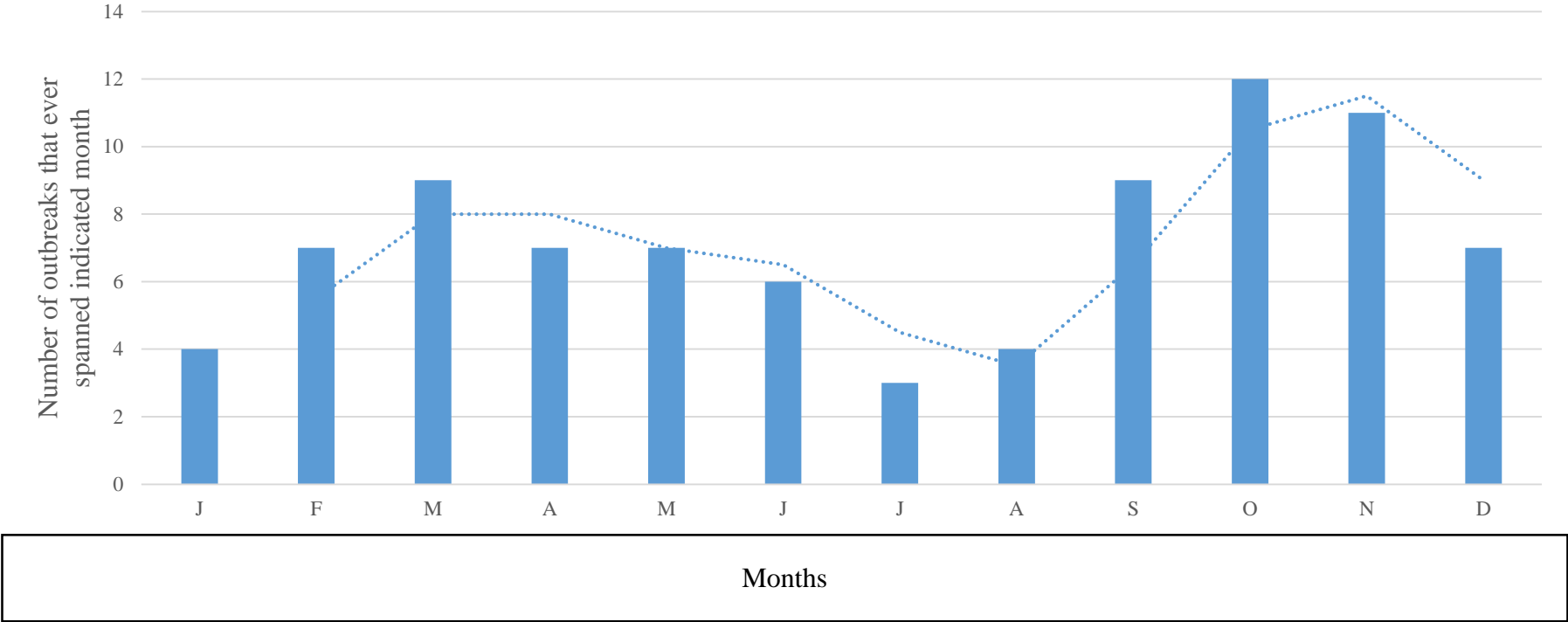

Supplement: S2 Fig — [5,8,9,17,20–22,31–55]. (PDF) [file pntd.0010233.s002.pdf]
