# Supplementary material for: Clinical manifestations of Rift Valley fever in humans: Systematic review and meta-analysis
Source: PLoS Negl Trop Dis. 2022 Mar 25;16(3):e0010233. doi: 10.1371/journal.pntd.0010233 (PMC8986116; doi:10.1371/journal.pntd.0010233)
Supplement: S5 Fig — n–number of patients with the sign or symptom; N–total number of patients in the study assessed for sign or symptom; %—percentage; ES (95% CI)–estimated 95% confidence interval; % weight–percentage weight of the study calculated from random effects meta-analysis; I2 –chi-square value; p–p-value; Inpatients–subjects source in the study was hospital based patients requiring admission; Outpatients–subjects source in the study was hospital based patients requiring no admission; Inpatients and outpatients -subjects source in the study was both hospital based patients requiring admission and no admission and data collection in the included studies was combined; Community patients -subjects source in the study was non-hospital based patients found in the community or at home. [5,17,21,22,31,32,34,40,41,44,46,49,50]. (PDF) [file pntd.0010233.s005.pdf]

S5 Fig. Forest plots for the common symptoms under the hepatic syndrome

a. Proportion of RVF patients with liver failure

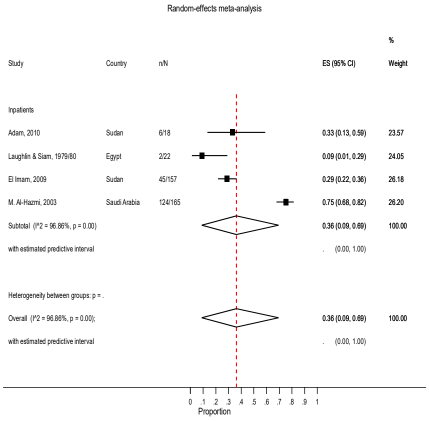

b. Proportion of RVF patients with right hypochondriac tenderness

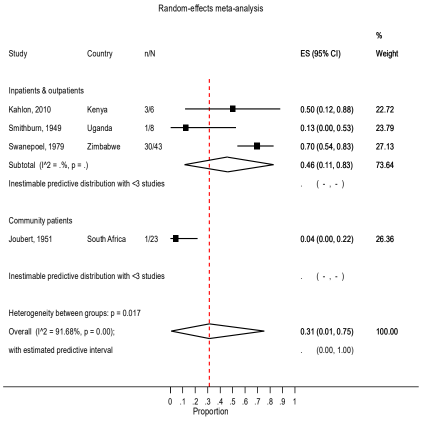

c. Proportion of RVF patients with hepatomegaly

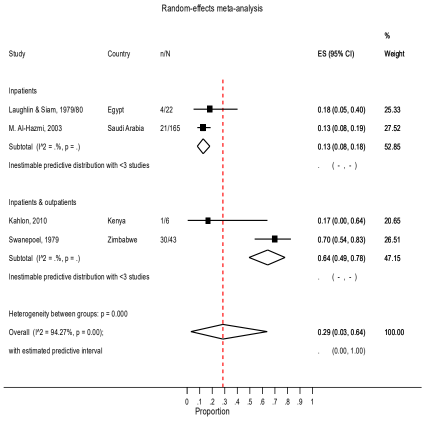

d. Proportion of RVF patients with splenomegaly

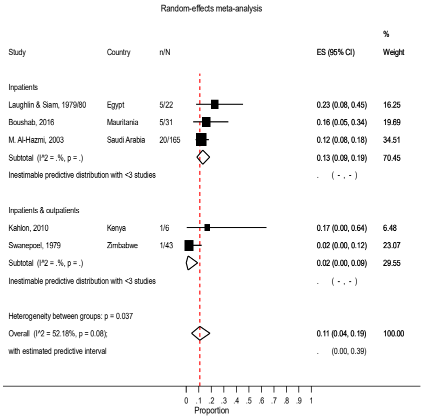

e Proportion of RVF patients with elevated aspartate aminotransferase(AST)

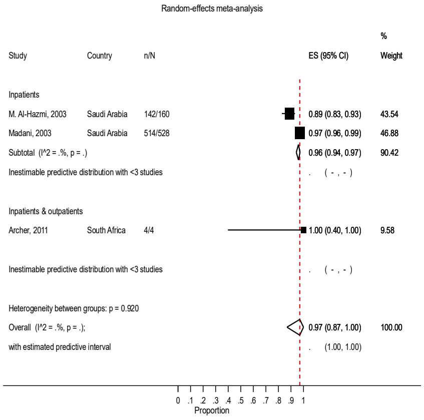

f. Proportion of RVF patients with elevated alanine aminotransferase(ALT)

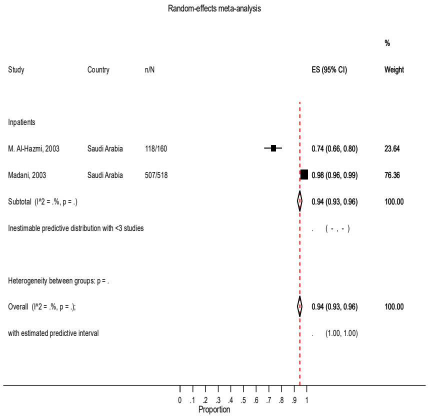

g. Proportion of RVF patients with elevated lactate dehydrogenase (LDH)

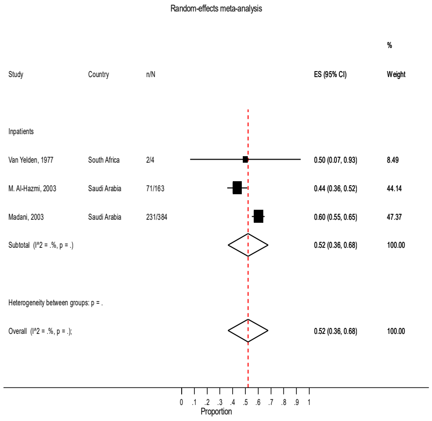

This is the S5 fig legend

n – number of patients with the sign or symptom; N – total number of patients in the study assessed for sign or symptom; % - percentage; ES (95% CI) – estimated 95% confidence interval; % weight – percentage weight of the study calculated from random effects meta-analysis; I^2 – chi-square value; p – p-value; Inpatients – subjects source in the study was hospital based patients requiring admission; Outpatients – subjects source in the study was hospital based patients requiring no admission; Inpatients and outpatients - subjects source in the study was both hospital based patients requiring admission and no admission and data collection in the included studies was combined; Community patients - subjects source in the study was non-hospital based patients found in the community or at home.
