# Supplementary material for: Clinical manifestations of Rift Valley fever in humans: Systematic review and meta-analysis
Source: PLoS Negl Trop Dis. 2022 Mar 25;16(3):e0010233. doi: 10.1371/journal.pntd.0010233 (PMC8986116; doi:10.1371/journal.pntd.0010233)
Supplement: S6 Fig — n–number of patients with the sign or symptom; N–total number of patients in the study assessed for sign or symptom; %—percentage; ES (95% CI)–estimated 95% confidence interval; % weight–percentage weight of the study calculated from random effects meta-analysis; I2– chi-square value; p–p-value; Inpatients–subjects source in the study was hospital bas ed patients requiring admission; Outpatients–subjects source in the study was hospital based patients requiring no admission; Inpatients and outpatients—subjects source in the study was both hospital based patients requiring admission and no admission and data collection in the included studies was combined; Community patients -subjects source in the study was non-hospital based patients found in the community or at home. [5,21,31,32]. (PDF) [file pntd.0010233.s006.pdf]

S6 Fig. Forest plots for the common symptoms under the renal syndrome

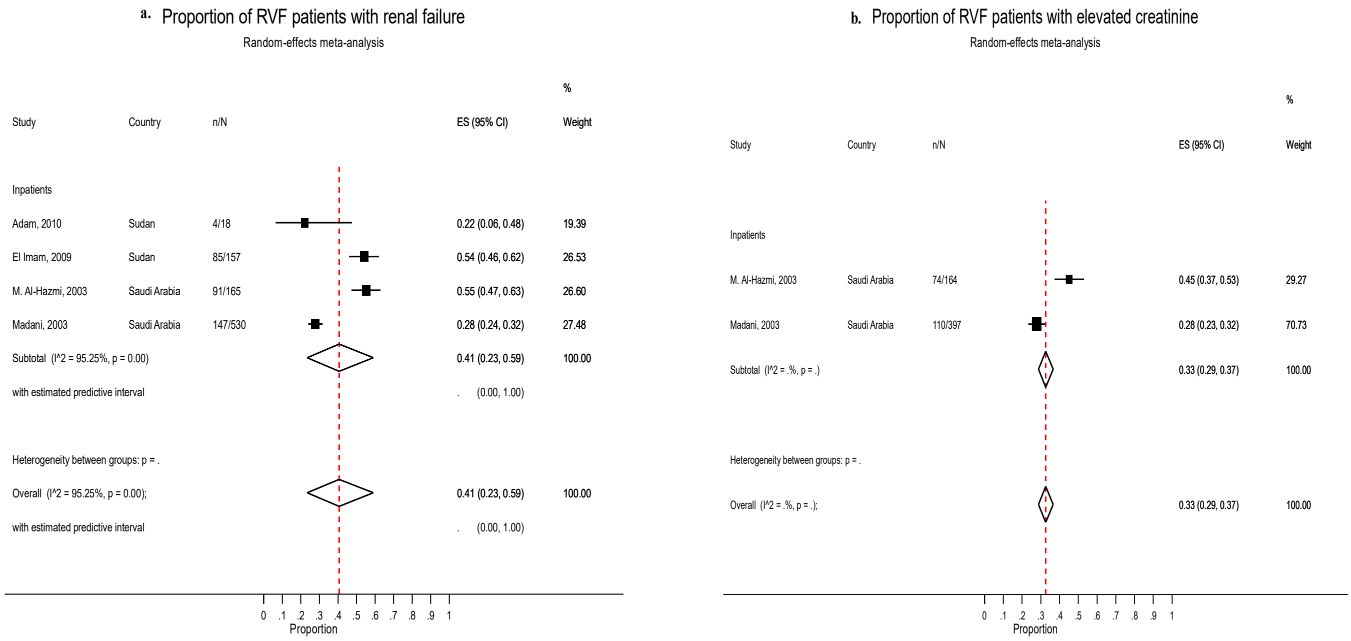

This is the S6 fig legend

n – number of patients with the sign or symptom; N – total number of patients in the study assessed for sign or symptom; % - percentage; ES (95% CI) – estimated 95% confidence interval; % weight – percentage weight of the study calculated from random effects meta-analysis; I^2 – chi-square value; p – p-value; Inpatients – subjects source in the study was hospital based patients requiring admission; Outpatients – subjects source in the study was hospital based patients requiring no admission; Inpatients and outpatients - subjects source in the study was both hospital based patients requiring admission and no admission and data collection in the included studies was combined; Community patients - subjects source in the study was non-hospital based patients found in the community or at home.
