# Supplementary material for: Clinical manifestations of Rift Valley fever in humans: Systematic review and meta-analysis
Source: PLoS Negl Trop Dis. 2022 Mar 25;16(3):e0010233. doi: 10.1371/journal.pntd.0010233 (PMC8986116; doi:10.1371/journal.pntd.0010233)
Supplement: S1 Table — (DOCX) [file pntd.0010233.s011.docx]

**S1 Table. Search strategy in Embase database**

| **[No](https://ovidsp.dc1.ovid.com/sp-4.02.1a/ovidweb.cgi?&S=IPFHFPBJCFACMFENKPCKLGGJHMINAA00&Sort+Sets=descending).** | **Searches** | **Results** |
| --- | --- | --- |
| 1 | Rift Valley fever.mp. [mp=title, abstract, heading word, drug trade name, original title, device manufacturer, drug manufacturer, device trade name, keyword, floating subheading word, candidate term word] | 2384 |
| 2 | Bunyaviridae.mp. [mp=title, abstract, heading word, drug trade name, original title, device manufacturer, drug manufacturer, device trade name, keyword, floating subheading word, candidate term word] | 1940 |
| 3 | Phlebovirus.mp. [mp=title, abstract, heading word, drug trade name, original title, device manufacturer, drug manufacturer, device trade name, keyword, floating subheading word, candidate term word] | 911 |
| 4 | exp Rift Valley fever/ | 848 |
| 5 | exp Bunyavirus/ or exp Bunyaviridae/ | 8128 |
| 6 | 1 or 2 or 3 or 4 or 5 | 10523 |
| 7 | (clinical adj1 (manifestation* or feature* or presentation*)).mp. [mp=title, abstract, heading word, drug trade name, original title, device manufacturer, drug manufacturer, device trade name, keyword, floating subheading word, candidate term word] | 876819 |
| 8 | (sign* or symptom* or morbidity or mortality or death or sequelae).mp. [mp=title, abstract, heading word, drug trade name, original title, device manufacturer, drug manufacturer, device trade name, keyword, floating subheading word, candidate term word] | 12249258 |
|  |  |  |
| 9 | ((complication* or long term complication* or long-term complication*) adj3 (liver or hepatic or abdominal or eye or visual or ocular or h?ematolog* or h?emorrhag* or bleeding or coagulation or clotting or cardiovascular or blood or brain or central nervous system or encephaliti*)).mp. [mp=title, abstract, heading word, drug trade name, original title, device manufacturer, drug manufacturer, device trade name, keyword, floating subheading word, candidate term word] | 99383 |
| 10 | (abortion or miscarriage or pregnancy loss).mp. [mp=title, abstract, heading word, drug trade name, original title, device manufacturer, drug manufacturer, device trade name, keyword, floating subheading word, candidate term word] | 134610 |
| 11 | (laboratory abnormalities or full blood count or FBC or complete blood count or CBC or leuco* or neutrop* or lympho* or eosinop* or basop* or monocyt* or haemoglobin or haematocrit or platelets or prothrombin time).mp. [mp=title, abstract, heading word, drug trade name, original title, device manufacturer, drug manufacturer, device trade name, keyword, floating subheading word, candidate term word] | 2311142 |
| 12 | (liver function tests or LFTs or bilirubin or aspartate aminotransferase or AST or serum glutamic oxaloacetic transaminase or SGOT or alanine aminotransferase or ALT or serum glutamic pyruvic transaminase or SGPT or alkaline phosphatase or ALP or gamma-glutamyl transpeptidase or GGT or albumin or total protein).mp. [mp=title, abstract, heading word, drug trade name, original title, device manufacturer, drug manufacturer, device trade name, keyword, floating subheading word, candidate term word] | 665247 |
| 13 | (renal function tests or RFTs or creatinine or serum creatinine or urea or blood urea nitrogen or BUN or glomerular filtration rate or GFR).mp. [mp=title, abstract, heading word, drug trade name, original title, device manufacturer, drug manufacturer, device trade name, keyword, floating subheading word, candidate term word] | 441122 |
| 14 | 7 or 8 or 9 or 10 or 11 or 12 or 13 | 14440333 |
| 15 | exp Africa/ | 337713 |
| 16 | (Africa south of the Sahara or Sub-Saharan Africa or Central Africa or East* Africa or Southern Africa or West* Africa or North* Africa).ti,ab. | 49231 |
| 17 | 15 or 16 | 348944 |
| 18 | exp Angola/ | 1417 |
| 19 | Angola.ti,ab. | 1525 |
| 20 | 18 or 19 | 1847 |
| 21 | exp Algeria/ | 4819 |
| 22 | Algeria.ti,ab. | 4760 |
| 23 | 21 or 22 | 5915 |
| 24 | exp Benin/ | 2400 |
| 25 | (Benin or Dahomey).ti,ab. | 4607 |
| 26 | 24 or 25 | 4902 |
| 27 | exp Botswana/ | 2599 |
| 28 | Botswana.ti,ab. | 2596 |
| 29 | Bechuanaland.ti,ab. | 34 |
| 30 | 27 or 28 or 29 | 3077 |
| 31 | exp Burkina Faso/ | 4115 |
| 32 | (Burkina Faso or Burkina Fasso or Upper Volta).ti,ab. | 4775 |
| 33 | 31 or 32 | 5353 |
| 34 | exp Burundi/ | 827 |
| 35 | Burundi.ti,ab. | 859 |
| 36 | 34 or 35 | 1041 |
| 37 | exp Cameroon/ | 6888 |
| 38 | Cameroon.ti,ab. | 7555 |
| 39 | 37 or 38 | 8706 |
| 40 | exp Cape Verde/ | 341 |
| 41 | Cape Verde.ti,ab. | 515 |
| 42 | 40 or 41 | 602 |
| 43 | exp Central African Republic/ | 916 |
| 44 | Central African Republic.ti,ab. | 1088 |
| 45 | Ubangi-Shari.ti,ab. | 1 |
| 46 | 43 or 44 or 45 | 1353 |
| 47 | exp Chad/ | 986 |
| 48 | Chad.ti,ab. | 1372 |
| 49 | 47 or 48 | 1725 |
| 50 | exp Comoros/ | 319 |
| 51 | (Comoro Islands or Iles Comores or Mayotte).ti,ab. | 339 |
| 52 | 50 or 51 | 562 |
| 53 | exp Congo/ | 3927 |
| 54 | Congo.ti,ab. | 15389 |
| 55 | Congo- Brazzaville.ti,ab. | 138 |
| 56 | 53 or 54 or 55 | 16332 |
| 57 | exp Cote d'Ivoire/ | 3242 |
| 58 | (Cote d'Ivoire or Ivory Coast).ti,ab. | 4262 |
| 59 | 57 or 58 | 5045 |
| 60 | exp Democratic Republic Congo/ | 3975 |
| 61 | (Democratic Republic of Congo or Belgian Congo or Zaire or Congo-Kinshasa).ti,ab. | 4767 |
| 62 | 60 or 61 | 6397 |
| 63 | exp Djibouti/ | 334 |
| 64 | Djibouti.ti,ab. | 413 |
| 65 | 63 or 64 | 521 |
| 66 | exp Egypt/ | 20560 |
| 67 | Egypt.ti,ab. | 18152 |
| 68 | 66 or 67 | 25514 |
| 69 | exp Guinea/ | 2650 |
| 70 | (Guinea not (Guinea pig or Guinea fowl or Guinea worm or Guinea grass or Papua New Guinea)).ti,ab. | 50866 |
| 71 | 69 or 70 | 51753 |
| 72 | exp Equatorial Guinea/ | 446 |
| 73 | Equatorial Guinea.ti,ab. | 537 |
| 74 | 72 or 73 | 629 |
| 75 | exp Eritrea/ | 544 |
| 76 | Eritrea.ti,ab. | 635 |
| 77 | 75 or 76 | 754 |
| 78 | exp Ethiopia/ | 15486 |
| 79 | Ethiopia.ti,ab. | 14722 |
| 80 | 78 or 79 | 17507 |
| 81 | exp Gabon/ | 1701 |
| 82 | (Gabon or Gabonese Republic).ti,ab. | 1870 |
| 83 | 81 or 82 | 2294 |
| 84 | exp Gambia/ | 2772 |
| 85 | (Gambia or The Gambia).ti,ab. | 2495 |
| 86 | 84 or 85 | 3389 |
| 87 | exp Ghana/ | 11086 |
| 88 | (Ghana or Gold Coast).ti,ab. | 11762 |
| 89 | 87 or 88 | 13498 |
| 90 | exp Guinea-Bissau/ | 1063 |
| 91 | (Guinea-Bissau or Portuguese Guinea).ti,ab. | 1100 |
| 92 | 90 or 91 | 1368 |
| 93 | exp Kenya/ | 20873 |
| 94 | Kenya.ti,ab. | 20190 |
| 95 | 93 or 94 | 24528 |
| 96 | exp Lesotho/ | 680 |
| 97 | (Lesotho or Basutoland).ti,ab. | 746 |
| 98 | 96 or 97 | 883 |
| 99 | exp Liberia/ | 1730 |
| 100 | Liberia.ti,ab. | 1729 |
| 101 | 99 or 100 | 2255 |
| 102 | exp Libyan Arab Jamahiriya/ | 1686 |
| 103 | Libya.ti,ab. | 1479 |
| 104 | 102 or 103 | 2072 |
| 105 | exp Madagascar/ | 4509 |
| 106 | (Madagascar or Malagasy Republic).ti,ab. | 5171 |
| 107 | 105 or 106 | 5878 |
| 108 | exp Malawi/ | 7075 |
| 109 | (Malawi or Nyasaland).ti,ab. | 7449 |
| 110 | 108 or 109 | 8497 |
| 111 | exp Mali/ | 3387 |
| 112 | Mali.ti,ab. | 4193 |
| 113 | 111 or 112 | 4837 |
| 114 | exp Mauritania/ | 620 |
| 115 | Mauritania.ti,ab. | 651 |
| 116 | 114 or 115 | 831 |
| 117 | exp Mauritius/ | 881 |
| 118 | Mauritius.ti,ab. | 1020 |
| 119 | 117 or 118 | 1269 |
| 120 | exp Morocco/ | 7592 |
| 121 | Morocco.ti,ab. | 7055 |
| 122 | 120 or 121 | 9269 |
| 123 | exp Mozambique/ | 3573 |
| 124 | (Mozambique or Portuguese East Africa).ti,ab. | 3823 |
| 125 | 123 or 124 | 4468 |
| 126 | exp Namibia/ | 1517 |
| 127 | (Namibia or Kalahari).ti,ab. | 1763 |
| 128 | 126 or 127 | 2134 |
| 129 | exp Niger/ | 2351 |
| 130 | Niger.ti,ab. | 16268 |
| 131 | 129 or 130 | 16601 |
| 132 | exp Nigeria/ | 37566 |
| 133 | Nigeria.ti,ab. | 34651 |
| 134 | 132 or 133 | 43770 |
| 135 | exp Rwanda/ | 3455 |
| 136 | (Rwanda or Ruanda).ti,ab. | 3420 |
| 137 | 135 or 136 | 4153 |
| 138 | exp "Sao Tome and Principe"/ | 74 |
| 139 | "Sao Tome and Principe".ti,ab. | 149 |
| 140 | 138 or 139 | 179 |
| 141 | exp Senegal/ | 6860 |
| 142 | Senegal.ti,ab. | 6690 |
| 143 | 141 or 142 | 8689 |
| 144 | exp Seychelles/ | 421 |
| 145 | Seychelles.ti,ab. | 706 |
| 146 | 144 or 145 | 774 |
| 147 | exp Sierra Leone/ | 2243 |
| 148 | Sierra Leone.ti,ab. | 2377 |
| 149 | 147 or 148 | 2892 |
| 150 | exp Somalia/ | 1972 |
| 151 | Somalia.ti,ab. | 1420 |
| 152 | 150 or 151 | 2398 |
| 153 | exp South Africa/ | 50316 |
| 154 | South Africa.ti,ab. | 38208 |
| 155 | 153 or 154 | 58813 |
| 156 | exp South Sudan/ | 245 |
| 157 | South Sudan.ti,ab. | 562 |
| 158 | 156 or 157 | 609 |
| 159 | exp Sudan/ | 7185 |
| 160 | Sudan.ti,ab. | 10235 |
| 161 | 159 or 160 | 11806 |
| 162 | exp Swaziland/ | 916 |
| 163 | Swaziland.ti,ab. | 934 |
| 164 | 162 or 163 | 1129 |
| 165 | exp Tanzania/ | 14795 |
| 166 | Tanzania.ti,ab. | 13909 |
| 167 | (Tanganyika or Zanzibar).ti,ab. | 1256 |
| 168 | 165 or 166 or 167 | 17225 |
| 169 | exp Togo/ | 1417 |
| 170 | (Togo or Togolese Republic).ti,ab. | 1630 |
| 171 | 169 or 170 | 1933 |
| 172 | exp Tunisia/ | 9666 |
| 173 | Tunisia.ti,ab. | 8209 |
| 174 | 172 or 173 | 11535 |
| 175 | exp Uganda/ | 17663 |
| 176 | Uganda.ti,ab. | 16620 |
| 177 | 175 or 176 | 20323 |
| 178 | Western Sahara.ti,ab. | 59 |
| 179 | Western Sahara.ti,ab. | 59 |
| 180 | 178 or 179 | 59 |
| 181 | exp Zambia/ | 6096 |
| 182 | (Zambia or Northern Rhodesia).ti,ab. | 5789 |
| 183 | 181 or 182 | 7099 |
| 184 | exp Zimbabwe/ | 6844 |
| 185 | (Zimbabwe or Rhodesia).ti,ab. | 6280 |
| 186 | 184 or 185 | 8168 |
| 187 | exp Middle East/ | 179190 |
| 188 | Arabian Peninsula.ti,ab. | 999 |
| 189 | 187 or 188 | 179752 |
| 190 | exp Saudi Arabia/ | 20065 |
| 191 | Saudi Arabia.ti,ab. | 18389 |
| 192 | 190 or 191 | 23680 |
| 193 | exp Jordan/ | 6346 |
| 194 | Jordan.ti,ab. | 7558 |
| 195 | 193 or 194 | 8932 |
| 196 | exp Iran/ | 47778 |
| 197 | Iran.ti,ab. | 49447 |
| 198 | 196 or 197 | 60501 |
| 199 | exp Iraq/ | 8235 |
| 200 | Iraq.ti,ab. | 8848 |
| 201 | 199 or 200 | 11619 |
| 202 | exp Kuwait/ | 4810 |
| 203 | Kuwait.ti,ab. | 4843 |
| 204 | 202 or 203 | 5824 |
| 205 | exp Bahrain/ | 1371 |
| 206 | Bahrain.ti,ab. | 1453 |
| 207 | 205 or 206 | 1801 |
| 208 | exp Qatar/ | 2579 |
| 209 | Qatar.ti,ab. | 2693 |
| 210 | 208 or 209 | 3121 |
| 211 | exp United Arab Emirates/ | 3455 |
| 212 | United Arab Emirates.ti,ab. | 2751 |
| 213 | 211 or 212 | 3986 |
| 214 | exp Oman/ | 2601 |
| 215 | Oman.ti,ab. | 2780 |
| 216 | 214 or 215 | 3268 |
| 217 | exp Yemen/ | 1981 |
| 218 | Yemen.ti,ab. | 2034 |
| 219 | 217 or 218 | 2561 |
| 220 | 17 or 20 or 23 or 26 or 30 or 33 or 36 or 39 or 42 or 46 or 49 or 52 or 56 or 59 or 62 or 65 or 68 or 71 or 74 or 77 or 80 or 83 or 86 or 89 or 92 or 95 or 98 or 101 or 104 or 107 or 110 or 113 or 116 or 119 or 122 or 125 or 128 or 131 or 134 or 137 or 140 or 143 or 146 or 149 or 152 or 155 or 158 or 161 or 164 or 168 or 171 or 174 or 177 or 180 or 183 or 186 or 189 or 192 or 195 or 198 or 201 or 204 or 207 or 210 or 213 or 216 or 219 | 654504 |
| 221 | 6 and 14 and 220 | 932 |
|  | **Search done on 13th October 2019** |  |
