# Supplementary material for: Clinical manifestations of Rift Valley fever in humans: Systematic review and meta-analysis
Source: PLoS Negl Trop Dis. 2022 Mar 25;16(3):e0010233. doi: 10.1371/journal.pntd.0010233 (PMC8986116; doi:10.1371/journal.pntd.0010233)
Supplement: S2 Table — (DOCX) [file pntd.0010233.s012.docx]

**S2 Table. Search strategy in Medline database**

| **No.** | **Searches** | **Results** |
| --- | --- | --- |
| 1 | Rift Valley fever.mp. [mp=title, abstract, original title, name of substance word, subject heading word, floating sub-heading word, keyword heading word, organism supplementary concept word, protocol supplementary concept word, rare disease supplementary concept word, unique identifier, synonyms] | 1738 |
| 2 | Bunyaviridae.mp. [mp=title, abstract, original title, name of substance word, subject heading word, floating sub-heading word, keyword heading word, organism supplementary concept word, protocol supplementary concept word, rare disease supplementary concept word, unique identifier, synonyms] | 2917 |
| 3 | Phlebovirus.mp. [mp=title, abstract, original title, name of substance word, subject heading word, floating sub-heading word, keyword heading word, organism supplementary concept word, protocol supplementary concept word, rare disease supplementary concept word, unique identifier, synonyms] | 904 |
| 4 | exp Rift Valley Fever/ | 1120 |
| 5 | exp Bunyaviridae Infections/ or exp Bunyaviridae/ | 10833 |
| 6 | 1 or 2 or 3 or 4 or 5 | 11238 |
| 7 | (clinical adj1 (manifestation* or feature* or presentation*)).mp. [mp=title, abstract, original title, name of substance word, subject heading word, floating sub-heading word, keyword heading word, organism supplementary concept word, protocol supplementary concept word, rare disease supplementary concept word, unique identifier, synonyms] | 197052 |
| 8 | (sign* or symptom* or morbidity or mortality or death or sequelae).mp. [mp=title, abstract, original title, name of substance word, subject heading word, floating sub-heading word, keyword heading word, organism supplementary concept word, protocol supplementary concept word, rare disease supplementary concept word, unique identifier, synonyms] | 7623668 |
| 9 | ((complication* or long term complication* or long-term complication*) adj3 (liver or hepatic or abdominal or eye or visual or ocular or h?ematolog* or h?emorrhag* or bleeding or coagulation or clotting or cardiovascular or blood or brain or central nervous system or encephaliti*)).mp. [mp=title, abstract, original title, name of substance word, subject heading word, floating sub-heading word, keyword heading word, organism supplementary concept word, protocol supplementary concept word, rare disease supplementary concept word, unique identifier, synonyms] | 80417 |
| 10 | (abortion or miscarriage or pregnancy loss).mp. [mp=title, abstract, original title, name of substance word, subject heading word, floating sub-heading word, keyword heading word, organism supplementary concept word, protocol supplementary concept word, rare disease supplementary concept word, unique identifier, synonyms] | 87554 |
| 11 | 7 or 8 or 9 or 10 | 7813407 |
| 12 | (laboratory abnormalities or full blood count or FBC or complete blood count or CBC or leuco* or neutrop* or lympho* or eosinop* or basop* or monocyt* or haemoglobin or haematocrit or platelets or prothrombin time).mp. [mp=title, abstract, original title, name of substance word, subject heading word, floating sub-heading word, keyword heading word, organism supplementary concept word, protocol supplementary concept word, rare disease supplementary concept word, unique identifier, synonyms] | 1419755 |
| 13 | (liver function tests or LFTs or bilirubin or aspartate aminotransferase or AST or serum glutamic oxaloacetic transaminase or SGOT or alanine aminotransferase or ALT or serum glutamic pyruvic transaminase or SGPT or alkaline phosphatase or ALP or gamma-glutamyl transpeptidase or GGT or albumin or total protein).mp. [mp=title, abstract, original title, name of substance word, subject heading word, floating sub-heading word, keyword heading word, organism supplementary concept word, protocol supplementary concept word, rare disease supplementary concept word, unique identifier, synonyms] | 365728 |
| 14 | (renal function tests or RFTs or creatinine or serum creatinine or urea or blood urea nitrogen or BUN or glomerular filtration rate or GFR).mp. [mp=title, abstract, original title, name of substance word, subject heading word, floating sub-heading word, keyword heading word, organism supplementary concept word, protocol supplementary concept word, rare disease supplementary concept word, unique identifier, synonyms] | 235305 |
| 15 | 12 or 13 or 14 | 1940949 |
| 16 | exp Africa/ | 253429 |
| 17 | (Africa south of the Sahara or Sub-Saharan Africa or Central Africa or East* Africa or Southern Africa or West* Africa or North* Africa).ti,ab. | 34437 |
| 18 | 16 or 17 | 261511 |
| 19 | exp Angola/ | 947 |
| 20 | Angola.ti,ab. | 1158 |
| 21 | 19 or 20 | 1379 |
| 22 | exp Algeria/ | 2926 |
| 23 | Algeria.ti,ab. | 2552 |
| 24 | 22 or 23 | 3563 |
| 25 | exp Benin/ | 1473 |
| 26 | (Benin or Dahomey).ti,ab. | 2762 |
| 27 | 25 or 26 | 2978 |
| 28 | exp Botswana/ | 1665 |
| 29 | Botswana.ti,ab. | 1807 |
| 30 | Bechuanaland.ti,ab. | 17 |
| 31 | 28 or 29 or 30 | 2128 |
| 32 | exp Burkina Faso/ | 3065 |
| 33 | (Burkina Faso or Burkina Fasso or Upper Volta).ti,ab. | 3448 |
| 34 | 32 or 33 | 3892 |
| 35 | exp Burundi/ | 624 |
| 36 | Burundi.ti,ab. | 674 |
| 37 | 35 or 36 | 851 |
| 38 | exp Cameroon/ | 5198 |
| 39 | Cameroon.ti,ab. | 5436 |
| 40 | 38 or 39 | 6548 |
| 41 | exp Cabo Verde/ | 185 |
| 42 | Cape Verde.ti,ab. | 431 |
| 43 | 41 or 42 | 474 |
| 44 | exp Central African Republic/ | 763 |
| 45 | Central African Republic.ti,ab. | 877 |
| 46 | Ubangi-Shari.ti,ab. | 2 |
| 47 | 44 or 45 or 46 | 1075 |
| 48 | exp Chad/ | 694 |
| 49 | Chad.ti,ab. | 968 |
| 50 | 48 or 49 | 1123 |
| 51 | exp Comoros/ | 294 |
| 52 | (Comoro Islands or Iles Comores or Mayotte).ti,ab. | 273 |
| 53 | 51 or 52 | 420 |
| 54 | exp Congo/ | 1774 |
| 55 | Congo.ti,ab. | 9612 |
| 56 | Congo- Brazzaville.ti,ab. | 101 |
| 57 | 54 or 55 or 56 | 10278 |
| 58 | exp Cote d'Ivoire/ | 3014 |
| 59 | (Cote d'Ivoire or Ivory Coast).ti,ab. | 3259 |
| 60 | 58 or 59 | 3958 |
| 61 | exp "Democratic Republic of the Congo"/ | 3952 |
| 62 | (Democratic Republic of Congo or Belgian Congo or Zaire or Congo-Kinshasa).ti,ab. | 3649 |
| 63 | 61 or 62 | 5272 |
| 64 | exp Djibouti/ | 217 |
| 65 | Djibouti.ti,ab. | 327 |
| 66 | 64 or 65 | 372 |
| 67 | exp Egypt/ | 14062 |
| 68 | Egypt.ti,ab. | 10672 |
| 69 | 67 or 68 | 17406 |
| 70 | exp Guinea/ | 981 |
| 71 | (Guinea not (Guinea pig or Guinea fowl or Guinea worm or Guinea grass or Papua New Guinea)).ti,ab. | 31448 |
| 72 | 70 or 71 | 31672 |
| 73 | exp Equatorial Guinea/ | 250 |
| 74 | Equatorial Guinea.ti,ab. | 357 |
| 75 | 73 or 74 | 417 |
| 76 | exp Eritrea/ | 317 |
| 77 | Eritrea.ti,ab. | 419 |
| 78 | 76 or 77 | 500 |
| 79 | exp Ethiopia/ | 11440 |
| 80 | Ethiopia.ti,ab. | 10373 |
| 81 | 79 or 80 | 12887 |
| 82 | exp Gabon/ | 1414 |
| 83 | (Gabon or Gabonese Republic).ti,ab. | 1486 |
| 84 | 82 or 83 | 1872 |
| 85 | exp Gambia/ | 2341 |
| 86 | (Gambia or The Gambia).ti,ab. | 2008 |
| 87 | 85 or 86 | 2888 |
| 88 | exp Ghana/ | 7500 |
| 89 | (Ghana or Gold Coast).ti,ab. | 7656 |
| 90 | 88 or 89 | 9089 |
| 91 | exp Guinea-Bissau/ | 899 |
| 92 | (Guinea-Bissau or Portuguese Guinea).ti,ab. | 897 |
| 93 | 91 or 92 | 1117 |
| 94 | exp Kenya/ | 15074 |
| 95 | Kenya.ti,ab. | 14209 |
| 96 | 94 or 95 | 18171 |
| 97 | exp Lesotho/ | 398 |
| 98 | (Lesotho or Basutoland).ti,ab. | 555 |
| 99 | 97 or 98 | 610 |
| 100 | exp Liberia/ | 1151 |
| 101 | Liberia.ti,ab. | 1247 |
| 102 | 100 or 101 | 1608 |
| 103 | exp Libya/ | 1099 |
| 104 | Libya.ti,ab. | 949 |
| 105 | 103 or 104 | 1398 |
| 106 | exp Madagascar/ | 3273 |
| 107 | (Madagascar or Malagasy Republic).ti,ab. | 3897 |
| 108 | 106 or 107 | 4478 |
| 109 | exp Malawi/ | 4878 |
| 110 | (Malawi or Nyasaland).ti,ab. | 5259 |
| 111 | 109 or 110 | 6074 |
| 112 | exp Mali/ | 2259 |
| 113 | Mali.ti,ab. | 2880 |
| 114 | 112 or 113 | 3406 |
| 115 | exp Mauritania/ | 423 |
| 116 | Mauritania.ti,ab. | 511 |
| 117 | 115 or 116 | 613 |
| 118 | exp Mauritius/ | 538 |
| 119 | Mauritius.ti,ab. | 762 |
| 120 | 118 or 119 | 871 |
| 121 | exp Morocco/ | 5462 |
| 122 | Morocco.ti,ab. | 4361 |
| 123 | 121 or 122 | 6710 |
| 124 | exp Mozambique/ | 2226 |
| 125 | (Mozambique or Portuguese East Africa).ti,ab. | 2743 |
| 126 | 124 or 125 | 3124 |
| 127 | exp Namibia/ | 1006 |
| 128 | Namibia.ti,ab. | 1152 |
| 129 | 127 or 128 | 1422 |
| 130 | exp Niger/ | 1143 |
| 131 | Niger.ti,ab. | 10080 |
| 132 | 130 or 131 | 10273 |
| 133 | exp Nigeria/ | 27276 |
| 134 | Nigeria.ti,ab. | 21457 |
| 135 | 133 or 134 | 30259 |
| 136 | exp Rwanda/ | 2243 |
| 137 | (Rwanda or Ruanda).ti,ab. | 2310 |
| 138 | 136 or 137 | 2815 |
| 139 | exp "Sao Tome and Principe"/ | 12 |
| 140 | "Sao Tome and Principe".ti,ab. | 119 |
| 141 | 139 or 140 | 123 |
| 142 | exp Senegal/ | 5538 |
| 143 | Senegal.ti,ab. | 4890 |
| 144 | 142 or 143 | 6946 |
| 145 | exp Seychelles/ | 354 |
| 146 | Seychelles.ti,ab. | 581 |
| 147 | 145 or 146 | 619 |
| 148 | exp Sierra Leone/ | 1431 |
| 149 | Sierra Leone.ti,ab. | 1673 |
| 150 | 148 or 149 | 1961 |
| 151 | exp Somalia/ | 1509 |
| 152 | Somalia.ti,ab. | 1102 |
| 153 | 151 or 152 | 1945 |
| 154 | exp South Africa/ | 39909 |
| 155 | South Africa.ti,ab. | 25664 |
| 156 | 154 or 155 | 45655 |
| 157 | exp South Sudan/ | 129 |
| 158 | South Sudan.ti,ab. | 374 |
| 159 | 157 or 158 | 389 |
| 160 | exp Sudan/ | 4566 |
| 161 | Sudan.ti,ab. | 6448 |
| 162 | 160 or 161 | 7786 |
| 163 | exp Swaziland/ | 533 |
| 164 | Swaziland.ti,ab. | 695 |
| 165 | 163 or 164 | 771 |
| 166 | exp Tanzania/ | 10657 |
| 167 | Tanzania.ti,ab. | 9946 |
| 168 | (Tanganyika or Zanzibar).ti,ab. | 916 |
| 169 | 166 or 167 or 168 | 12745 |
| 170 | exp Togo/ | 1101 |
| 171 | (Togo or Togolese Republic).ti,ab. | 1240 |
| 172 | 170 or 171 | 1463 |
| 173 | exp Tunisia/ | 7926 |
| 174 | Tunisia.ti,ab. | 5512 |
| 175 | 173 or 174 | 9190 |
| 176 | exp Uganda/ | 11280 |
| 177 | Uganda.ti,ab. | 10950 |
| 178 | 176 or 177 | 13427 |
| 179 | Western Sahara.mp. | 52 |
| 180 | Western Sahara.ti,ab. | 52 |
| 181 | 179 or 180 | 52 |
| 182 | exp Zambia/ | 4265 |
| 183 | (Zambia or Northern Rhodesia).ti,ab. | 4187 |
| 184 | 182 or 183 | 5235 |
| 185 | exp Zimbabwe/ | 5591 |
| 186 | (Zimbabwe or Rhodesia).ti,ab. | 5062 |
| 187 | 185 or 186 | 6786 |
| 188 | exp Middle East/ | 126908 |
| 189 | Arabian Peninsula.ti,ab. | 704 |
| 190 | 188 or 189 | 127252 |
| 191 | exp Saudi Arabia/ | 12185 |
| 192 | Saudi Arabia.ti,ab. | 10232 |
| 193 | 191 or 192 | 14340 |
| 194 | exp Jordan/ | 3924 |
| 195 | Jordan.ti,ab. | 4422 |
| 196 | 194 or 195 | 5521 |
| 197 | exp Iran/ | 24503 |
| 198 | Iran.ti,ab. | 19058 |
| 199 | 197 or 198 | 28254 |
| 200 | exp Iraq/ | 4458 |
| 201 | Iraq.ti,ab. | 5280 |
| 202 | 200 or 201 | 7323 |
| 203 | exp Kuwait/ | 2966 |
| 204 | Kuwait.ti,ab. | 2811 |
| 205 | 203 or 204 | 3621 |
| 206 | exp Bahrain/ | 574 |
| 207 | Bahrain.ti,ab. | 661 |
| 208 | 206 or 207 | 801 |
| 209 | exp Qatar/ | 1032 |
| 210 | Qatar.ti,ab. | 1096 |
| 211 | 209 or 210 | 1326 |
| 212 | exp United Arab Emirates/ | 1896 |
| 213 | United Arab Emirates.ti,ab. | 1672 |
| 214 | 212 or 213 | 2300 |
| 215 | exp Oman/ | 1385 |
| 216 | Oman.ti,ab. | 1545 |
| 217 | 215 or 216 | 1885 |
| 218 | exp Yemen/ | 1328 |
| 219 | Yemen.ti,ab. | 1384 |
| 220 | 218 or 219 | 1788 |
| 221 | 11 or 15 | 8930247 |
| 222 | 18 or 21 or 24 or 27 or 31 or 34 or 37 or 40 or 43 or 47 or 50 or 53 or 57 or 60 or 63 or 66 or 69 or 72 or 75 or 78 or 81 or 84 or 87 or 90 or 93 or 96 or 99 or 102 or 105 or 108 or 111 or 114 or 117 or 120 or 123 or 126 or 129 or 132 or 135 or 138 or 141 or 144 or 147 or 150 or 153 or 156 or 159 or 162 or 165 or 169 or 172 or 175 or 178 or 181 or 184 or 187 or 190 or 193 or 196 or 199 or 202 or 205 or 208 or 211 or 214 or 217 or 220 | 462558 |
| 223 | 6 and 221 and 222 | 940 |
|  | **Search done on 14th October 2019** |  |
