# Supplementary material for: Clinical manifestations of Rift Valley fever in humans: Systematic review and meta-analysis
Source: PLoS Negl Trop Dis. 2022 Mar 25;16(3):e0010233. doi: 10.1371/journal.pntd.0010233 (PMC8986116; doi:10.1371/journal.pntd.0010233)
Supplement: S3 Table — (DOCX) [file pntd.0010233.s013.docx]

**S3 Table. Search strategy in Global Health database**

| No. | **Searches** | **Results** |
| --- | --- | --- |
| 1 | Rift Valley fever.mp. [mp=abstract, title, original title, broad terms, heading words, identifiers, cabicodes] | 2116 |
| 2 | Bunyaviridae.mp. [mp=abstract, title, original title, broad terms, heading words, identifiers, cabicodes] | 9566 |
| 3 | Phlebovirus.mp. [mp=abstract, title, original title, broad terms, heading words, identifiers, cabicodes] | 2826 |
| 4 | exp Rift Valley fever/ | 981 |
| 5 | exp Bunyaviridae/ | 10057 |
| 6 | 1 or 2 or 3 or 4 or 5 | 10211 |
| 7 | (clinical adj1 (manifestation* or feature* or presentation*)).mp. [mp=abstract, title, original title, broad terms, heading words, identifiers, cabicodes] | 41839 |
| 8 | (sign* or symptom* or morbidity or mortality or death or sequelae).mp. [mp=abstract, title, original title, broad terms, heading words, identifiers, cabicodes] | 1444605 |
| 9 | ((complication* or long term complication* or long-term complication*) adj3 (liver or hepatic or abdominal or eye or visual or ocular or h?ematolog* or h?emorrhag* or bleeding or coagulation or clotting or cardiovascular or blood or brain or central nervous system or encephaliti*)).mp. [mp=abstract, title, original title, broad terms, heading words, identifiers, cabicodes] | 5571 |
| 10 | (abortion or miscarriage or pregnancy loss).mp. [mp=abstract, title, original title, broad terms, heading words, identifiers, cabicodes] | 14326 |
| 11 | (laboratory abnormalities or full blood count or FBC or complete blood count or CBC or leuco* or neutrop* or lympho* or eosinop* or basop* or monocyt* or haemoglobin or haematocrit or platelets or prothrombin time).mp. [mp=abstract, title, original title, broad terms, heading words, identifiers, cabicodes] | 241357 |
| 12 | (liver function tests or LFTs or bilirubin or aspartate aminotransferase or AST or serum glutamic oxaloacetic transaminase or SGOT or alanine aminotransferase or ALT or serum glutamic pyruvic transaminase or SGPT or alkaline phosphatase or ALP or gamma-glutamyl transpeptidase or GGT or albumin or total protein).mp. [mp=abstract, title, original title, broad terms, heading words, identifiers, cabicodes] | 78014 |
| 13 | (renal function tests or RFTs or creatinine or serum creatinine or urea or blood urea nitrogen or BUN or glomerular filtration rate or GFR).mp. [mp=abstract, title, original title, broad terms, heading words, identifiers, cabicodes] | 43715 |
| 14 | 7 or 8 or 9 or 10 or 11 or 12 or 13 | 1635038 |
| 15 | exp Africa/ | 255046 |
| 16 | (Africa south of the Sahara or Sub-Saharan Africa or Central Africa or East* Africa or Southern Africa or West* Africa or North* Africa).ti,ab. | 33244 |
| 17 | 15 or 16 | 258598 |
| 18 | exp Angola/ | 1432 |
| 19 | Angola.ti,ab. | 1493 |
| 20 | 18 or 19 | 1624 |
| 21 | exp Algeria/ | 4645 |
| 22 | Algeria.ti,ab. | 3525 |
| 23 | 21 or 22 | 4868 |
| 24 | exp Benin/ | 2557 |
| 25 | (Benin or Dahomey).ti,ab. | 3387 |
| 26 | 24 or 25 | 3596 |
| 27 | exp Botswana/ | 1919 |
| 28 | Botswana.ti,ab. | 1692 |
| 29 | 27 or 28 | 2011 |
| 30 | exp Burkina Faso/ | 4747 |
| 31 | (Burkina Faso or Burkina Fasso or Upper Volta).ti,ab. | 4708 |
| 32 | 30 or 31 | 5113 |
| 33 | exp Burundi/ | 687 |
| 34 | Burundi.ti,ab. | 737 |
| 35 | 33 or 34 | 801 |
| 36 | exp Cameroon/ | 7186 |
| 37 | Cameroon.ti,ab. | 5929 |
| 38 | 36 or 37 | 7727 |
| 39 | exp Cape Verde/ | 312 |
| 40 | Cape Verde.ti,ab. | 320 |
| 41 | 39 or 40 | 348 |
| 42 | exp Central African Republic/ | 1091 |
| 43 | Central African Republic.ti,ab. | 1111 |
| 44 | Ubangi-Shari.ti,ab. | 5 |
| 45 | 42 or 43 or 44 | 1242 |
| 46 | exp Chad/ | 1217 |
| 47 | Chad.ti,ab. | 1249 |
| 48 | 46 or 47 | 1402 |
| 49 | exp Comoros/ | 302 |
| 50 | (Comoro Islands or Iles Comores or Mayotte).ti,ab. | 287 |
| 51 | 49 or 50 | 481 |
| 52 | exp Congo/ | 2166 |
| 53 | Congo.ti,ab. | 9422 |
| 54 | Congo- Brazzaville.ti,ab. | 136 |
| 55 | 52 or 53 or 54 | 9911 |
| 56 | exp Cote d'Ivoire/ | 4585 |
| 57 | (Cote d'Ivoire or Ivory Coast).ti,ab. | 4519 |
| 58 | 56 or 57 | 5040 |
| 59 | Congo Democratic Republic.gl. | 6987 |
| 60 | (Democratic Republic of Congo or Belgian Congo or Zaire or Congo-Kinshasa).ti,ab. | 5595 |
| 61 | 59 or 60 | 7677 |
| 62 | exp Djibouti/ | 342 |
| 63 | Djibouti.ti,ab. | 331 |
| 64 | 62 or 63 | 394 |
| 65 | exp Egypt/ | 18637 |
| 66 | Egypt.ti,ab. | 12613 |
| 67 | 65 or 66 | 19501 |
| 68 | exp Guinea/ | 1180 |
| 69 | (Guinea not (Guinea pig or Guinea fowl or Guinea worm or Guinea grass or Papua New Guinea)).ti,ab. | 10712 |
| 70 | 68 or 69 | 10872 |
| 71 | exp Equatorial Guinea/ | 415 |
| 72 | Equatorial Guinea.ti,ab. | 387 |
| 73 | 71 or 72 | 505 |
| 74 | exp Eritrea/ | 670 |
| 75 | Eritrea.ti,ab. | 644 |
| 76 | 74 or 75 | 735 |
| 77 | exp Ethiopia/ | 12848 |
| 78 | Ethiopia.ti,ab. | 11816 |
| 79 | 77 or 78 | 13277 |
| 80 | exp Gabon/ | 1705 |
| 81 | (Gabon or Gabonese Republic).ti,ab. | 1613 |
| 82 | 80 or 81 | 1863 |
| 83 | exp Gambia/ | 2920 |
| 84 | (Gambia or The Gambia).ti,ab. | 2530 |
| 85 | 83 or 84 | 3280 |
| 86 | exp Ghana/ | 9879 |
| 87 | (Ghana or Gold Coast).ti,ab. | 9336 |
| 88 | 86 or 87 | 10505 |
| 89 | exp Guinea-Bissau/ | 991 |
| 90 | (Guinea-Bissau or Portuguese Guinea).ti,ab. | 976 |
| 91 | 89 or 90 | 1088 |
| 92 | exp Kenya/ | 17664 |
| 93 | Kenya.ti,ab. | 15981 |
| 94 | 92 or 93 | 18522 |
| 95 | exp Lesotho/ | 608 |
| 96 | (Lesotho or Basutoland).ti,ab. | 603 |
| 97 | 95 or 96 | 667 |
| 98 | exp Liberia/ | 1630 |
| 99 | Liberia.ti,ab. | 1700 |
| 100 | 98 or 99 | 1859 |
| 101 | exp Libya/ | 1402 |
| 102 | Libya.ti,ab. | 945 |
| 103 | 101 or 102 | 1494 |
| 104 | exp Madagascar/ | 3846 |
| 105 | (Madagascar or Malagasy Republic).ti,ab. | 3830 |
| 106 | 104 or 105 | 4193 |
| 107 | exp Malawi/ | 6179 |
| 108 | (Malawi or Nyasaland).ti,ab. | 5732 |
| 109 | 107 or 108 | 6404 |
| 110 | exp Mali/ | 3120 |
| 111 | Mali.ti,ab. | 3040 |
| 112 | 110 or 111 | 3598 |
| 113 | exp Mauritania/ | 527 |
| 114 | Mauritania.ti,ab. | 584 |
| 115 | 113 or 114 | 637 |
| 116 | exp Mauritius/ | 957 |
| 117 | Mauritius.ti,ab. | 936 |
| 118 | 116 or 117 | 1040 |
| 119 | exp Morocco/ | 6999 |
| 120 | Morocco.ti,ab. | 4789 |
| 121 | 119 or 120 | 7334 |
| 122 | exp Mozambique/ | 3029 |
| 123 | (Mozambique or Portuguese East Africa).ti,ab. | 3016 |
| 124 | 122 or 123 | 3306 |
| 125 | exp Namibia/ | 1054 |
| 126 | (Namibia or Kalahari).ti,ab. | 931 |
| 127 | 125 or 126 | 1201 |
| 128 | exp Niger/ | 1296 |
| 129 | Niger.ti,ab. | 11385 |
| 130 | 128 or 129 | 11565 |
| 131 | exp Nigeria/ | 33517 |
| 132 | Nigeria.ti,ab. | 28472 |
| 133 | 131 or 132 | 34688 |
| 134 | exp Rwanda/ | 2269 |
| 135 | (Rwanda or Ruanda).ti,ab. | 2267 |
| 136 | 134 or 135 | 2551 |
| 137 | exp "Sao Tome and Principe"/ | 222 |
| 138 | "Sao Tome and Principe".ti,ab. | 117 |
| 139 | 137 or 138 | 239 |
| 140 | exp Senegal/ | 6426 |
| 141 | Senegal.ti,ab. | 5474 |
| 142 | 140 or 141 | 7076 |
| 143 | exp Seychelles/ | 331 |
| 144 | Seychelles.ti,ab. | 354 |
| 145 | 143 or 144 | 380 |
| 146 | exp Sierra Leone/ | 2151 |
| 147 | Sierra Leone.ti,ab. | 2167 |
| 148 | 146 or 147 | 2354 |
| 149 | exp Somalia/ | 1688 |
| 150 | Somalia.ti,ab. | 1212 |
| 151 | 149 or 150 | 1900 |
| 152 | exp South Africa/ | 31913 |
| 153 | South Africa.ti,ab. | 22270 |
| 154 | 152 or 153 | 33019 |
| 155 | exp South Sudan/ | 323 |
| 156 | South Sudan.ti,ab. | 440 |
| 157 | 155 or 156 | 470 |
| 158 | exp Sudan/ | 6815 |
| 159 | Sudan.ti,ab. | 7740 |
| 160 | 158 or 159 | 8876 |
| 161 | exp Swaziland/ | 799 |
| 162 | Swaziland.ti,ab. | 795 |
| 163 | 161 or 162 | 856 |
| 164 | exp Tanzania/ | 13309 |
| 165 | Tanzania.ti,ab. | 10438 |
| 166 | (Tanganyika or Zanzibar).ti,ab. | 2494 |
| 167 | 164 or 165 or 166 | 13889 |
| 168 | exp Togo/ | 1665 |
| 169 | (Togo or Togolese Republic).ti,ab. | 1512 |
| 170 | 168 or 169 | 1840 |
| 171 | exp Tunisia/ | 6513 |
| 172 | Tunisia.ti,ab. | 4040 |
| 173 | 171 or 172 | 6815 |
| 174 | exp Uganda/ | 13174 |
| 175 | Uganda.ti,ab. | 12449 |
| 176 | 174 or 175 | 13858 |
| 177 | exp Western Sahara/ | 28 |
| 178 | Western Sahara.ti,ab. | 31 |
| 179 | 177 or 178 | 39 |
| 180 | exp Zambia/ | 5128 |
| 181 | (Zambia or Northern Rhodesia).ti,ab. | 4590 |
| 182 | 180 or 181 | 5351 |
| 183 | exp Zimbabwe/ | 6516 |
| 184 | (Zimbabwe or Rhodesia).ti,ab. | 6177 |
| 185 | 183 or 184 | 6888 |
| 186 | exp Middle East/ | 103368 |
| 187 | Arabian Peninsula.ti,ab. | 445 |
| 188 | 186 or 187 | 103545 |
| 189 | exp Saudi Arabia/ | 9785 |
| 190 | Saudi Arabia.ti,ab. | 7611 |
| 191 | 189 or 190 | 9961 |
| 192 | exp Jordan/ | 3068 |
| 193 | Jordan.ti,ab. | 3356 |
| 194 | 192 or 193 | 3947 |
| 195 | exp Iran/ | 46590 |
| 196 | Iran.ti,ab. | 28108 |
| 197 | 195 or 196 | 47967 |
| 198 | exp Iraq/ | 4478 |
| 199 | Iraq.ti,ab. | 3503 |
| 200 | 198 or 199 | 4967 |
| 201 | exp Kuwait/ | 1918 |
| 202 | Kuwait.ti,ab. | 1598 |
| 203 | 201 or 202 | 2042 |
| 204 | exp Bahrain/ | 549 |
| 205 | Bahrain.ti,ab. | 460 |
| 206 | 204 or 205 | 579 |
| 207 | exp Qatar/ | 783 |
| 208 | Qatar.ti,ab. | 645 |
| 209 | 207 or 208 | 828 |
| 210 | exp United Arab Emirates/ | 1373 |
| 211 | United Arab Emirates.ti,ab. | 994 |
| 212 | 210 or 211 | 1424 |
| 213 | exp Oman/ | 1281 |
| 214 | Oman.ti,ab. | 1077 |
| 215 | 213 or 214 | 1369 |
| 216 | exp Yemen/ | 1654 |
| 217 | Yemen.ti,ab. | 1522 |
| 218 | 216 or 217 | 1827 |
| 219 | 17 or 20 or 23 or 26 or 29 or 32 or 35 or 38 or 41 or 45 or 48 or 51 or 55 or 58 or 61 or 64 or 67 or 70 or 73 or 76 or 79 or 82 or 85 or 88 or 91 or 94 or 97 or 100 or 103 or 106 or 109 or 112 or 115 or 118 or 121 or 124 or 127 or 130 or 133 or 136 or 139 or 142 or 145 or 148 or 151 or 154 or 157 or 160 or 163 or 167 or 170 or 173 or 176 or 179 or 182 or 185 or 188 or 191 or 194 or 197 or 200 or 203 or 206 or 209 or 212 or 215 or 218 | 367341 |
| 220 | 6 and 14 and 219 | 1258 |
|  | **Search done on 15th October 2019** |  |
