# Supplementary material for: Clinical manifestations of Rift Valley fever in humans: Systematic review and meta-analysis
Source: PLoS Negl Trop Dis. 2022 Mar 25;16(3):e0010233. doi: 10.1371/journal.pntd.0010233 (PMC8986116; doi:10.1371/journal.pntd.0010233)
Supplement: S4 Table — (DOCX) [file pntd.0010233.s014.docx]

**S4 Table. Search strategy in Web of Science database**

| **No.** | **Results** | **Searches** |
| --- | --- | --- |
|  | **Search done on 15th October 2019** | |
| # 78 | [635](https://apps.webofknowledge.com/summary.do?product=WOS&doc=1&qid=79&SID=E1U3gPrr8n13jdmywRj&search_mode=CombineSearches&update_back2search_link_param=yes) | #77 AND #9 AND #1 |
|  |  | Indexes=SCI-EXPANDED, SSCI, A&HCI, CPCI-S, CPCI-SSH, ESCI Timespan=All years |
| # 77 | [880,813](https://apps.webofknowledge.com/summary.do?product=WOS&doc=1&qid=78&SID=E1U3gPrr8n13jdmywRj&search_mode=CombineSearches&update_back2search_link_param=yes) | #76 OR #75 OR #74 OR #73 OR #72 OR #71 OR #70 OR #69 OR #68 OR #67 OR #66 OR #65 OR #64 OR #63 OR #62 OR #61 OR #60 OR #59 OR #58 OR #57 OR #56 OR #55 OR #54 OR #53 OR #52 OR #51 OR #50 OR #49 OR #48 OR #47 OR #46 OR #45 OR #44 OR #43 OR #42 OR #41 OR #40 OR #39 OR #38 OR #37 OR #36 OR #35 OR #34 OR #33 OR #32 OR #31 OR #30 OR #29 OR #28 OR #27 OR #26 OR #25 OR #24 OR #23 OR #22 OR #21 OR #20 OR #19 OR #18 OR #17 OR #16 OR #15 OR #14 OR #13 OR #12 OR #11 OR #10 |
|  |  | Indexes=SCI-EXPANDED, SSCI, A&HCI, CPCI-S, CPCI-SSH, ESCI Timespan=All years |
| # 76 | [4,230](https://apps.webofknowledge.com/summary.do?product=WOS&doc=1&qid=77&SID=E1U3gPrr8n13jdmywRj&search_mode=AdvancedSearch&update_back2search_link_param=yes) | ts=(Yemen) |
|  |  | Indexes=SCI-EXPANDED, SSCI, A&HCI, CPCI-S, CPCI-SSH, ESCI Timespan=All years |
| # 75 | [9,408](https://apps.webofknowledge.com/summary.do?product=WOS&doc=1&qid=76&SID=E1U3gPrr8n13jdmywRj&search_mode=AdvancedSearch&update_back2search_link_param=yes) | ts=(Oman) |
|  |  | Indexes=SCI-EXPANDED, SSCI, A&HCI, CPCI-S, CPCI-SSH, ESCI Timespan=All years |
| # 74 | [5,134](https://apps.webofknowledge.com/summary.do?product=WOS&doc=1&qid=75&SID=E1U3gPrr8n13jdmywRj&search_mode=AdvancedSearch&update_back2search_link_param=yes) | ts=(United Arab Emirates) |
|  |  | Indexes=SCI-EXPANDED, SSCI, A&HCI, CPCI-S, CPCI-SSH, ESCI Timespan=All years |
| # 73 | [3,790](https://apps.webofknowledge.com/summary.do?product=WOS&doc=1&qid=74&SID=E1U3gPrr8n13jdmywRj&search_mode=AdvancedSearch&update_back2search_link_param=yes) | ts=(Qatar) |
|  |  | Indexes=SCI-EXPANDED, SSCI, A&HCI, CPCI-S, CPCI-SSH, ESCI Timespan=All years |
| # 72 | [2,150](https://apps.webofknowledge.com/summary.do?product=WOS&doc=1&qid=73&SID=E1U3gPrr8n13jdmywRj&search_mode=AdvancedSearch&update_back2search_link_param=yes) | ts=(Bahrain) |
|  |  | Indexes=SCI-EXPANDED, SSCI, A&HCI, CPCI-S, CPCI-SSH, ESCI Timespan=All years |
| # 71 | [8,088](https://apps.webofknowledge.com/summary.do?product=WOS&doc=1&qid=72&SID=E1U3gPrr8n13jdmywRj&search_mode=AdvancedSearch&update_back2search_link_param=yes) | ts=(Kuwait) |
|  |  | Indexes=SCI-EXPANDED, SSCI, A&HCI, CPCI-S, CPCI-SSH, ESCI Timespan=All years |
| # 70 | [20,181](https://apps.webofknowledge.com/summary.do?product=WOS&doc=1&qid=71&SID=E1U3gPrr8n13jdmywRj&search_mode=AdvancedSearch&update_back2search_link_param=yes) | ts=(Iraq) |
|  |  | Indexes=SCI-EXPANDED, SSCI, A&HCI, CPCI-S, CPCI-SSH, ESCI Timespan=All years |
| # 69 | [81,861](https://apps.webofknowledge.com/summary.do?product=WOS&doc=1&qid=70&SID=E1U3gPrr8n13jdmywRj&search_mode=AdvancedSearch&update_back2search_link_param=yes) | ts=(Iran) |
|  |  | Indexes=SCI-EXPANDED, SSCI, A&HCI, CPCI-S, CPCI-SSH, ESCI Timespan=All years |
| # 68 | [25,947](https://apps.webofknowledge.com/summary.do?product=WOS&doc=1&qid=69&SID=E1U3gPrr8n13jdmywRj&search_mode=AdvancedSearch&update_back2search_link_param=yes) | ts=(Jordan) |
|  |  | Indexes=SCI-EXPANDED, SSCI, A&HCI, CPCI-S, CPCI-SSH, ESCI Timespan=All years |
| # 67 | [29,212](https://apps.webofknowledge.com/summary.do?product=WOS&doc=1&qid=68&SID=E1U3gPrr8n13jdmywRj&search_mode=AdvancedSearch&update_back2search_link_param=yes) | ts=(Saudi Arabia) |
|  |  | Indexes=SCI-EXPANDED, SSCI, A&HCI, CPCI-S, CPCI-SSH, ESCI Timespan=All years |
| # 66 | [2,932](https://apps.webofknowledge.com/summary.do?product=WOS&doc=1&qid=67&SID=E1U3gPrr8n13jdmywRj&search_mode=AdvancedSearch&update_back2search_link_param=yes) | ts=(Arabian Peninsula) |
|  |  | Indexes=SCI-EXPANDED, SSCI, A&HCI, CPCI-S, CPCI-SSH, ESCI Timespan=All years |
| # 65 | [46,743](https://apps.webofknowledge.com/summary.do?product=WOS&doc=1&qid=66&SID=E1U3gPrr8n13jdmywRj&search_mode=AdvancedSearch&update_back2search_link_param=yes) | ts=(Middle East) |
|  |  | Indexes=SCI-EXPANDED, SSCI, A&HCI, CPCI-S, CPCI-SSH, ESCI Timespan=All years |
| # 64 | [15,066](https://apps.webofknowledge.com/summary.do?product=WOS&doc=1&qid=65&SID=E1U3gPrr8n13jdmywRj&search_mode=AdvancedSearch&update_back2search_link_param=yes) | ts=(Zimbabwe or Rhodesia) |
|  |  | Indexes=SCI-EXPANDED, SSCI, A&HCI, CPCI-S, CPCI-SSH, ESCI Timespan=All years |
| # 63 | [9,871](https://apps.webofknowledge.com/summary.do?product=WOS&doc=1&qid=64&SID=E1U3gPrr8n13jdmywRj&search_mode=AdvancedSearch&update_back2search_link_param=yes) | ts=(Zambia or Northern Rhodesia) |
|  |  | Indexes=SCI-EXPANDED, SSCI, A&HCI, CPCI-S, CPCI-SSH, ESCI Timespan=All years |
| # 62 | [1,312](https://apps.webofknowledge.com/summary.do?product=WOS&doc=1&qid=63&SID=E1U3gPrr8n13jdmywRj&search_mode=AdvancedSearch&update_back2search_link_param=yes) | ts=(Western Sahara) |
|  |  | Indexes=SCI-EXPANDED, SSCI, A&HCI, CPCI-S, CPCI-SSH, ESCI Timespan=All years |
| # 61 | [2,245](https://apps.webofknowledge.com/summary.do?product=WOS&doc=1&qid=62&SID=E1U3gPrr8n13jdmywRj&search_mode=AdvancedSearch&update_back2search_link_param=yes) | ts=(Togo or Togolese Republic) |
|  |  | Indexes=SCI-EXPANDED, SSCI, A&HCI, CPCI-S, CPCI-SSH, ESCI Timespan=All years |
| # 60 | [23,640](https://apps.webofknowledge.com/summary.do?product=WOS&doc=1&qid=61&SID=E1U3gPrr8n13jdmywRj&search_mode=AdvancedSearch&update_back2search_link_param=yes) | ts=(Uganda) |
|  |  | Indexes=SCI-EXPANDED, SSCI, A&HCI, CPCI-S, CPCI-SSH, ESCI Timespan=All years |
| # 59 | [15,860](https://apps.webofknowledge.com/summary.do?product=WOS&doc=1&qid=60&SID=E1U3gPrr8n13jdmywRj&search_mode=AdvancedSearch&update_back2search_link_param=yes) | ts=(Tunisia) |
|  |  | Indexes=SCI-EXPANDED, SSCI, A&HCI, CPCI-S, CPCI-SSH, ESCI Timespan=All years |
| # 58 | [29,891](https://apps.webofknowledge.com/summary.do?product=WOS&doc=1&qid=59&SID=E1U3gPrr8n13jdmywRj&search_mode=AdvancedSearch&update_back2search_link_param=yes) | ts=(Tanzania or Tanganyika or Zanzibar) |
|  |  | Indexes=SCI-EXPANDED, SSCI, A&HCI, CPCI-S, CPCI-SSH, ESCI Timespan=All years |
| # 57 | [1,893](https://apps.webofknowledge.com/summary.do?product=WOS&doc=1&qid=58&SID=E1U3gPrr8n13jdmywRj&search_mode=AdvancedSearch&update_back2search_link_param=yes) | ts=(Swaziland) |
|  |  | Indexes=SCI-EXPANDED, SSCI, A&HCI, CPCI-S, CPCI-SSH, ESCI Timespan=All years |
| # 56 | [14,726](https://apps.webofknowledge.com/summary.do?product=WOS&doc=1&qid=57&SID=E1U3gPrr8n13jdmywRj&search_mode=AdvancedSearch&update_back2search_link_param=yes) | ts=(Sudan) |
|  |  | Indexes=SCI-EXPANDED, SSCI, A&HCI, CPCI-S, CPCI-SSH, ESCI Timespan=All years |
| # 55 | [1,720](https://apps.webofknowledge.com/summary.do?product=WOS&doc=1&qid=56&SID=E1U3gPrr8n13jdmywRj&search_mode=AdvancedSearch&update_back2search_link_param=yes) | ts=(South Sudan) |
|  |  | Indexes=SCI-EXPANDED, SSCI, A&HCI, CPCI-S, CPCI-SSH, ESCI Timespan=All years |
| # 54 | [128,792](https://apps.webofknowledge.com/summary.do?product=WOS&doc=1&qid=55&SID=E1U3gPrr8n13jdmywRj&search_mode=AdvancedSearch&update_back2search_link_param=yes) | ts=(South Africa) |
|  |  | Indexes=SCI-EXPANDED, SSCI, A&HCI, CPCI-S, CPCI-SSH, ESCI Timespan=All years |
| # 53 | [3,281](https://apps.webofknowledge.com/summary.do?product=WOS&doc=1&qid=54&SID=E1U3gPrr8n13jdmywRj&search_mode=AdvancedSearch&update_back2search_link_param=yes) | ts=(Somalia) |
|  |  | Indexes=SCI-EXPANDED, SSCI, A&HCI, CPCI-S, CPCI-SSH, ESCI Timespan=All years |
| # 52 | [4,523](https://apps.webofknowledge.com/summary.do?product=WOS&doc=1&qid=53&SID=E1U3gPrr8n13jdmywRj&search_mode=AdvancedSearch&update_back2search_link_param=yes) | ts=(Sierra Leone) |
|  |  | Indexes=SCI-EXPANDED, SSCI, A&HCI, CPCI-S, CPCI-SSH, ESCI Timespan=All years |
| # 51 | [2,360](https://apps.webofknowledge.com/summary.do?product=WOS&doc=1&qid=52&SID=E1U3gPrr8n13jdmywRj&search_mode=AdvancedSearch&update_back2search_link_param=yes) | ts=(Seychelles) |
|  |  | Indexes=SCI-EXPANDED, SSCI, A&HCI, CPCI-S, CPCI-SSH, ESCI Timespan=All years |
| # 50 | [10,425](https://apps.webofknowledge.com/summary.do?product=WOS&doc=1&qid=51&SID=E1U3gPrr8n13jdmywRj&search_mode=AdvancedSearch&update_back2search_link_param=yes) | ts=(Senegal) |
|  |  | Indexes=SCI-EXPANDED, SSCI, A&HCI, CPCI-S, CPCI-SSH, ESCI Timespan=All years |
| # 49 | [238](https://apps.webofknowledge.com/summary.do?product=WOS&doc=1&qid=50&SID=E1U3gPrr8n13jdmywRj&search_mode=AdvancedSearch&update_back2search_link_param=yes) | ts=("Sao Tome and Principe") |
|  |  | Indexes=SCI-EXPANDED, SSCI, A&HCI, CPCI-S, CPCI-SSH, ESCI Timespan=All years |
| # 48 | [6,054](https://apps.webofknowledge.com/summary.do?product=WOS&doc=1&qid=49&SID=E1U3gPrr8n13jdmywRj&search_mode=AdvancedSearch&update_back2search_link_param=yes) | ts=(Rwanda or Ruanda) |
|  |  | Indexes=SCI-EXPANDED, SSCI, A&HCI, CPCI-S, CPCI-SSH, ESCI Timespan=All years |
| # 47 | [47,653](https://apps.webofknowledge.com/summary.do?product=WOS&doc=1&qid=48&SID=E1U3gPrr8n13jdmywRj&search_mode=AdvancedSearch&update_back2search_link_param=yes) | ts=(Nigeria) |
|  |  | Indexes=SCI-EXPANDED, SSCI, A&HCI, CPCI-S, CPCI-SSH, ESCI Timespan=All years |
| # 46 | [31,516](https://apps.webofknowledge.com/summary.do?product=WOS&doc=1&qid=47&SID=E1U3gPrr8n13jdmywRj&search_mode=AdvancedSearch&update_back2search_link_param=yes) | ts=(Niger) |
|  |  | Indexes=SCI-EXPANDED, SSCI, A&HCI, CPCI-S, CPCI-SSH, ESCI Timespan=All years |
| # 45 | [9,385](https://apps.webofknowledge.com/summary.do?product=WOS&doc=1&qid=46&SID=E1U3gPrr8n13jdmywRj&search_mode=AdvancedSearch&update_back2search_link_param=yes) | ts=(Namibia or Kalahari) |
|  |  | Indexes=SCI-EXPANDED, SSCI, A&HCI, CPCI-S, CPCI-SSH, ESCI Timespan=All years |
| # 44 | [9,122](https://apps.webofknowledge.com/summary.do?product=WOS&doc=1&qid=45&SID=E1U3gPrr8n13jdmywRj&search_mode=AdvancedSearch&update_back2search_link_param=yes) | ts=(Mozambique or Portuguese East Africa) |
|  |  | Indexes=SCI-EXPANDED, SSCI, A&HCI, CPCI-S, CPCI-SSH, ESCI Timespan=All years |
| # 43 | [18,733](https://apps.webofknowledge.com/summary.do?product=WOS&doc=1&qid=44&SID=E1U3gPrr8n13jdmywRj&search_mode=AdvancedSearch&update_back2search_link_param=yes) | ts=(Morocco) |
|  |  | Indexes=SCI-EXPANDED, SSCI, A&HCI, CPCI-S, CPCI-SSH, ESCI Timespan=All years |
| # 42 | [3,057](https://apps.webofknowledge.com/summary.do?product=WOS&doc=1&qid=43&SID=E1U3gPrr8n13jdmywRj&search_mode=AdvancedSearch&update_back2search_link_param=yes) | ts=(Mauritius) |
|  |  | Indexes=SCI-EXPANDED, SSCI, A&HCI, CPCI-S, CPCI-SSH, ESCI Timespan=All years |
| # 41 | [1,793](https://apps.webofknowledge.com/summary.do?product=WOS&doc=1&qid=42&SID=E1U3gPrr8n13jdmywRj&search_mode=AdvancedSearch&update_back2search_link_param=yes) | ts=(Mauritania) |
|  |  | Indexes=SCI-EXPANDED, SSCI, A&HCI, CPCI-S, CPCI-SSH, ESCI Timespan=All years |
| # 40 | [7,082](https://apps.webofknowledge.com/summary.do?product=WOS&doc=1&qid=41&SID=E1U3gPrr8n13jdmywRj&search_mode=AdvancedSearch&update_back2search_link_param=yes) | ts=(Mali) |
|  |  | Indexes=SCI-EXPANDED, SSCI, A&HCI, CPCI-S, CPCI-SSH, ESCI Timespan=All years |
| # 39 | [12,531](https://apps.webofknowledge.com/summary.do?product=WOS&doc=1&qid=40&SID=E1U3gPrr8n13jdmywRj&search_mode=AdvancedSearch&update_back2search_link_param=yes) | ts=(Malawi or Nyasaland) |
|  |  | Indexes=SCI-EXPANDED, SSCI, A&HCI, CPCI-S, CPCI-SSH, ESCI Timespan=All years |
| # 38 | [13,429](https://apps.webofknowledge.com/summary.do?product=WOS&doc=1&qid=39&SID=E1U3gPrr8n13jdmywRj&search_mode=AdvancedSearch&update_back2search_link_param=yes) | ts=(Madagascar or Malagasy Republic) |
|  |  | Indexes=SCI-EXPANDED, SSCI, A&HCI, CPCI-S, CPCI-SSH, ESCI Timespan=All years |
| # 37 | [4,677](https://apps.webofknowledge.com/summary.do?product=WOS&doc=1&qid=38&SID=E1U3gPrr8n13jdmywRj&search_mode=AdvancedSearch&update_back2search_link_param=yes) | ts=(Libya) |
|  |  | Indexes=SCI-EXPANDED, SSCI, A&HCI, CPCI-S, CPCI-SSH, ESCI Timespan=All years |
| # 36 | [2,679](https://apps.webofknowledge.com/summary.do?product=WOS&doc=1&qid=37&SID=E1U3gPrr8n13jdmywRj&search_mode=AdvancedSearch&update_back2search_link_param=yes) | ts=(Liberia) |
|  |  | Indexes=SCI-EXPANDED, SSCI, A&HCI, CPCI-S, CPCI-SSH, ESCI Timespan=All years |
| # 35 | [2,003](https://apps.webofknowledge.com/summary.do?product=WOS&doc=1&qid=36&SID=E1U3gPrr8n13jdmywRj&search_mode=AdvancedSearch&update_back2search_link_param=yes) | ts=(Lesotho or Basutoland) |
|  |  | Indexes=SCI-EXPANDED, SSCI, A&HCI, CPCI-S, CPCI-SSH, ESCI Timespan=All years |
| # 34 | [38,717](https://apps.webofknowledge.com/summary.do?product=WOS&doc=1&qid=35&SID=E1U3gPrr8n13jdmywRj&search_mode=AdvancedSearch&update_back2search_link_param=yes) | ts=(Kenya) |
|  |  | Indexes=SCI-EXPANDED, SSCI, A&HCI, CPCI-S, CPCI-SSH, ESCI Timespan=All years |
| # 33 | [1,945](https://apps.webofknowledge.com/summary.do?product=WOS&doc=1&qid=34&SID=E1U3gPrr8n13jdmywRj&search_mode=AdvancedSearch&update_back2search_link_param=yes) | ts=(Guinea-Bissau or Portuguese Guinea) |
|  |  | Indexes=SCI-EXPANDED, SSCI, A&HCI, CPCI-S, CPCI-SSH, ESCI Timespan=All years |
| # 32 | [22,993](https://apps.webofknowledge.com/summary.do?product=WOS&doc=1&qid=33&SID=E1U3gPrr8n13jdmywRj&search_mode=AdvancedSearch&update_back2search_link_param=yes) | ts=(Ghana or Gold Coast) |
|  |  | Indexes=SCI-EXPANDED, SSCI, A&HCI, CPCI-S, CPCI-SSH, ESCI Timespan=All years |
| # 31 | [3,235](https://apps.webofknowledge.com/summary.do?product=WOS&doc=1&qid=32&SID=E1U3gPrr8n13jdmywRj&search_mode=AdvancedSearch&update_back2search_link_param=yes) | ts=(Gambia or The Gambia) |
|  |  | Indexes=SCI-EXPANDED, SSCI, A&HCI, CPCI-S, CPCI-SSH, ESCI Timespan=All years |
| # 30 | [3,379](https://apps.webofknowledge.com/summary.do?product=WOS&doc=1&qid=31&SID=E1U3gPrr8n13jdmywRj&search_mode=AdvancedSearch&update_back2search_link_param=yes) | ts=(Gabon or Gabonese Republic) |
|  |  | Indexes=SCI-EXPANDED, SSCI, A&HCI, CPCI-S, CPCI-SSH, ESCI Timespan=All years |
| # 29 | [26,514](https://apps.webofknowledge.com/summary.do?product=WOS&doc=1&qid=30&SID=E1U3gPrr8n13jdmywRj&search_mode=AdvancedSearch&update_back2search_link_param=yes) | ts=(Ethiopia) |
|  |  | Indexes=SCI-EXPANDED, SSCI, A&HCI, CPCI-S, CPCI-SSH, ESCI Timespan=All years |
| # 28 | [1,454](https://apps.webofknowledge.com/summary.do?product=WOS&doc=1&qid=29&SID=E1U3gPrr8n13jdmywRj&search_mode=AdvancedSearch&update_back2search_link_param=yes) | ts=(Eritrea) |
|  |  | Indexes=SCI-EXPANDED, SSCI, A&HCI, CPCI-S, CPCI-SSH, ESCI Timespan=All years |
| # 27 | [1,551](https://apps.webofknowledge.com/summary.do?product=WOS&doc=1&qid=28&SID=E1U3gPrr8n13jdmywRj&search_mode=AdvancedSearch&update_back2search_link_param=yes) | ts=(Equatorial Guinea) |
|  |  | Indexes=SCI-EXPANDED, SSCI, A&HCI, CPCI-S, CPCI-SSH, ESCI Timespan=All years |
| # 26 | [12,440](https://apps.webofknowledge.com/summary.do?product=WOS&doc=1&qid=27&SID=E1U3gPrr8n13jdmywRj&search_mode=AdvancedSearch&update_back2search_link_param=yes) | ts=(Guinea not (Guinea pig or Guinea fowl or Guinea worm or Guinea grass or Papua New Guinea)) |
|  |  | Indexes=SCI-EXPANDED, SSCI, A&HCI, CPCI-S, CPCI-SSH, ESCI Timespan=All years |
| # 25 | [39,759](https://apps.webofknowledge.com/summary.do?product=WOS&doc=1&qid=26&SID=E1U3gPrr8n13jdmywRj&search_mode=AdvancedSearch&update_back2search_link_param=yes) | ts=(Egypt) |
|  |  | Indexes=SCI-EXPANDED, SSCI, A&HCI, CPCI-S, CPCI-SSH, ESCI Timespan=All years |
| # 24 | [628](https://apps.webofknowledge.com/summary.do?product=WOS&doc=1&qid=25&SID=E1U3gPrr8n13jdmywRj&search_mode=AdvancedSearch&update_back2search_link_param=yes) | ts=(Djibouti) |
|  |  | Indexes=SCI-EXPANDED, SSCI, A&HCI, CPCI-S, CPCI-SSH, ESCI Timespan=All years |
| # 23 | [8,434](https://apps.webofknowledge.com/summary.do?product=WOS&doc=1&qid=24&SID=E1U3gPrr8n13jdmywRj&search_mode=AdvancedSearch&update_back2search_link_param=yes) | ts=(Democratic Republic of Congo or Belgian Congo or Zaire or Congo-Kinshasa) |
|  |  | Indexes=SCI-EXPANDED, SSCI, A&HCI, CPCI-S, CPCI-SSH, ESCI Timespan=All years |
| # 22 | [22,277](https://apps.webofknowledge.com/summary.do?product=WOS&doc=1&qid=23&SID=E1U3gPrr8n13jdmywRj&search_mode=AdvancedSearch&update_back2search_link_param=yes) | ts=(Congo or Congo- Brazzaville) |
|  |  | Indexes=SCI-EXPANDED, SSCI, A&HCI, CPCI-S, CPCI-SSH, ESCI Timespan=All years |
| # 21 | [1,167](https://apps.webofknowledge.com/summary.do?product=WOS&doc=1&qid=22&SID=E1U3gPrr8n13jdmywRj&search_mode=AdvancedSearch&update_back2search_link_param=yes) | ts=(Comoros or Comoro Islands or Iles Comores or Mayotte) |
|  |  | Indexes=SCI-EXPANDED, SSCI, A&HCI, CPCI-S, CPCI-SSH, ESCI Timespan=All years |
| # 20 | [2,609](https://apps.webofknowledge.com/summary.do?product=WOS&doc=1&qid=21&SID=E1U3gPrr8n13jdmywRj&search_mode=AdvancedSearch&update_back2search_link_param=yes) | ts=(Chad) |
|  |  | Indexes=SCI-EXPANDED, SSCI, A&HCI, CPCI-S, CPCI-SSH, ESCI Timespan=All years |
| # 19 | [1,695](https://apps.webofknowledge.com/summary.do?product=WOS&doc=1&qid=20&SID=E1U3gPrr8n13jdmywRj&search_mode=AdvancedSearch&update_back2search_link_param=yes) | ts=(Central African Republic or Ubangi-Shari) |
|  |  | Indexes=SCI-EXPANDED, SSCI, A&HCI, CPCI-S, CPCI-SSH, ESCI Timespan=All years |
| # 18 | [1,969](https://apps.webofknowledge.com/summary.do?product=WOS&doc=1&qid=19&SID=E1U3gPrr8n13jdmywRj&search_mode=AdvancedSearch&update_back2search_link_param=yes) | ts=(Cape Verde) |
|  |  | Indexes=SCI-EXPANDED, SSCI, A&HCI, CPCI-S, CPCI-SSH, ESCI Timespan=All years |
| # 17 | [13,354](https://apps.webofknowledge.com/summary.do?product=WOS&doc=1&qid=18&SID=E1U3gPrr8n13jdmywRj&search_mode=AdvancedSearch&update_back2search_link_param=yes) | ts=(Cameroon) |
|  |  | Indexes=SCI-EXPANDED, SSCI, A&HCI, CPCI-S, CPCI-SSH, ESCI Timespan=All years |
| # 16 | [1,645](https://apps.webofknowledge.com/summary.do?product=WOS&doc=1&qid=17&SID=E1U3gPrr8n13jdmywRj&search_mode=AdvancedSearch&update_back2search_link_param=yes) | ts=(Burundi) |
|  |  | Indexes=SCI-EXPANDED, SSCI, A&HCI, CPCI-S, CPCI-SSH, ESCI Timespan=All years |
| # 15 | [7,837](https://apps.webofknowledge.com/summary.do?product=WOS&doc=1&qid=16&SID=E1U3gPrr8n13jdmywRj&search_mode=AdvancedSearch&update_back2search_link_param=yes) | ts=(Burkina Faso or Burkina Fasso or Upper Volta) |
|  |  | Indexes=SCI-EXPANDED, SSCI, A&HCI, CPCI-S, CPCI-SSH, ESCI Timespan=All years |
| # 14 | [6,946](https://apps.webofknowledge.com/summary.do?product=WOS&doc=1&qid=15&SID=E1U3gPrr8n13jdmywRj&search_mode=AdvancedSearch&update_back2search_link_param=yes) | ts=(Botswana or Bechuanaland) |
|  |  | Indexes=SCI-EXPANDED, SSCI, A&HCI, CPCI-S, CPCI-SSH, ESCI Timespan=All years |
| # 13 | [5,980](https://apps.webofknowledge.com/summary.do?product=WOS&doc=1&qid=14&SID=E1U3gPrr8n13jdmywRj&search_mode=AdvancedSearch&update_back2search_link_param=yes) | ts=(Benin or Dahomey) |
|  |  | Indexes=SCI-EXPANDED, SSCI, A&HCI, CPCI-S, CPCI-SSH, ESCI Timespan=All years |
| # 12 | [13,520](https://apps.webofknowledge.com/summary.do?product=WOS&doc=1&qid=13&SID=E1U3gPrr8n13jdmywRj&search_mode=AdvancedSearch&update_back2search_link_param=yes) | ts=(Algeria) |
|  |  | Indexes=SCI-EXPANDED, SSCI, A&HCI, CPCI-S, CPCI-SSH, ESCI Timespan=All years |
| # 11 | [4,513](https://apps.webofknowledge.com/summary.do?product=WOS&doc=1&qid=12&SID=E1U3gPrr8n13jdmywRj&search_mode=AdvancedSearch&update_back2search_link_param=yes) | ts=(Angola) |
|  |  | Indexes=SCI-EXPANDED, SSCI, A&HCI, CPCI-S, CPCI-SSH, ESCI Timespan=All years |
| # 10 | [306,432](https://apps.webofknowledge.com/summary.do?product=WOS&doc=1&qid=11&SID=E1U3gPrr8n13jdmywRj&search_mode=AdvancedSearch&update_back2search_link_param=yes) | ts=(Africa) |
|  |  | Indexes=SCI-EXPANDED, SSCI, A&HCI, CPCI-S, CPCI-SSH, ESCI Timespan=All years |
| # 9 | [12,866,072](https://apps.webofknowledge.com/summary.do?product=WOS&doc=1&qid=10&SID=E1U3gPrr8n13jdmywRj&search_mode=CombineSearches&update_back2search_link_param=yes) | #8 OR #7 OR #6 OR #5 OR #4 OR #3 OR #2 |
|  |  | Indexes=SCI-EXPANDED, SSCI, A&HCI, CPCI-S, CPCI-SSH, ESCI Timespan=All years |
| # 8 | [243,530](https://apps.webofknowledge.com/summary.do?product=WOS&doc=1&qid=9&SID=E1U3gPrr8n13jdmywRj&search_mode=AdvancedSearch&update_back2search_link_param=yes) | ts=(renal function tests or RFTs or creatinine or serum creatinine or urea or blood urea nitrogen or BUN or glomerular filtration rate or GFR) |
|  |  | Indexes=SCI-EXPANDED, SSCI, A&HCI, CPCI-S, CPCI-SSH, ESCI Timespan=All years |
| # 7 | [602,582](https://apps.webofknowledge.com/summary.do?product=WOS&doc=1&qid=8&SID=E1U3gPrr8n13jdmywRj&search_mode=AdvancedSearch&update_back2search_link_param=yes) | ts=(liver function tests or LFTs or bilirubin or aspartate aminotransferase or AST or serum glutamic oxaloacetic transaminase or SGOT or alanine aminotransferase or ALT or serum glutamic pyruvic transaminase or SGPT or alkaline phosphatase or ALP or gamma-glutamyl transpeptidase or GGT or albumin or total protein) |
|  |  | Indexes=SCI-EXPANDED, SSCI, A&HCI, CPCI-S, CPCI-SSH, ESCI Timespan=All years |
| # 6 | [1,619,889](https://apps.webofknowledge.com/summary.do?product=WOS&doc=1&qid=6&SID=E1U3gPrr8n13jdmywRj&search_mode=AdvancedSearch&update_back2search_link_param=yes) | ts=(laboratory abnormalities or full blood count or FBC or complete blood count or CBC or leuco* or neutrop* or lympho* or eosinop* or basop* or monocyt* or haemoglobin or haematocrit or platelets or prothrombin time) |
|  |  | Indexes=SCI-EXPANDED, SSCI, A&HCI, CPCI-S, CPCI-SSH, ESCI Timespan=All years |
| # 5 | [80,610](https://apps.webofknowledge.com/summary.do?product=WOS&doc=1&qid=5&SID=E1U3gPrr8n13jdmywRj&search_mode=AdvancedSearch&update_back2search_link_param=yes) | ts=(abortion or miscarriage or pregnancy loss) |
|  |  | Indexes=SCI-EXPANDED, SSCI, A&HCI, CPCI-S, CPCI-SSH, ESCI Timespan=All years |
| # 4 | [297,294](https://apps.webofknowledge.com/summary.do?product=WOS&doc=1&qid=4&SID=E1U3gPrr8n13jdmywRj&search_mode=AdvancedSearch&update_back2search_link_param=yes) | ts=((complication* or long term complication* or long-term complication*) AND (liver or hepatic or abdominal or eye or visual or ocular or h?ematolog* or h?emorrhag* or bleeding or coagulation or clotting or cardiovascular or blood or brain or central nervous system or encephaliti*)) |
|  |  | Indexes=SCI-EXPANDED, SSCI, A&HCI, CPCI-S, CPCI-SSH, ESCI Timespan=All years |
| # 3 | [11,263,458](https://apps.webofknowledge.com/summary.do?product=WOS&doc=1&qid=3&SID=E1U3gPrr8n13jdmywRj&search_mode=AdvancedSearch&update_back2search_link_param=yes) | ts=(sign* or symptom* or morbidity or mortality or death or sequelae) |
|  |  | Indexes=SCI-EXPANDED, SSCI, A&HCI, CPCI-S, CPCI-SSH, ESCI Timespan=All years |
| # 2 | [202,361](https://apps.webofknowledge.com/summary.do?product=WOS&doc=1&qid=2&SID=E1U3gPrr8n13jdmywRj&search_mode=AdvancedSearch&update_back2search_link_param=yes) | ts=(clinical NEAR/1 (manifestation* or feature* or presentation*)) |
|  |  | Indexes=SCI-EXPANDED, SSCI, A&HCI, CPCI-S, CPCI-SSH, ESCI Timespan=All years |
| # 1 | [3,743](https://apps.webofknowledge.com/summary.do?product=WOS&doc=1&qid=1&SID=E1U3gPrr8n13jdmywRj&search_mode=AdvancedSearch&update_back2search_link_param=yes) | ts=(Rift Valley fever OR Rift Valley fever virus OR Bunyaviridae OR Phlebovirus) |
|  |  | Indexes=SCI-EXPANDED, SSCI, A&HCI, CPCI-S, CPCI-SSH, ESCI Timespan=All years |
