# Supplementary material for: Clinical manifestations of Rift Valley fever in humans: Systematic review and meta-analysis
Source: PLoS Negl Trop Dis. 2022 Mar 25;16(3):e0010233. doi: 10.1371/journal.pntd.0010233 (PMC8986116; doi:10.1371/journal.pntd.0010233)
Supplement: S5 Table — (DOCX) [file pntd.0010233.s015.docx]

**S5 Table. Risk of Bias Tool (adopted and modified from the Hoy and Brooks tool)**

| **Risk of bias item** | **Criteria for answers (please circle one option)** |
| --- | --- |
| **External validity** | |
| 1. Was selection bias minimal | - Yes (LOW RISK): The study’s population included an appropriate proportion of both male and female subjects of all ages - No (HIGH RISK): The study’s population DID NOT included an equal proportion of both male and female subjects or some age groups were not enrolled |
| 2. Was the likelihood of non-response bias minimal? | - Yes (LOW RISK): The response rate for the study was >/=75%, OR, an analysis was performed that showed no significant difference in relevant demographic characteristics between responders and non-responders - No (HIGH RISK): The response rate was <75%, and if any analysis comparing responders and non-responders was done, it showed a significant difference in relevant demographic characteristics between responders and non-responders. |
| **Internal validity** | |
| 3. Were data collected directly from the subjects (as opposed to a proxy)? | - Yes (LOW RISK): All data were collected directly from the subjects. - No (HIGH RISK): In some instances, data were collected from a proxy. |
| 4. Was there a case definition used in the study? | - Yes (LOW RISK): A case definition was used. - No (HIGH RISK): A case definition was NOT used. |
| 5. Was the study instrument that measured the parameter of interest (e.g. prevalence of different symptoms) shown to have reliability and validity (if necessary)? | - Yes (LOW RISK): The study instrument had been shown to have reliability and validity (if this was necessary), e.g. test-re- test, piloting, validation in a previous study, etc. - No (HIGH RISK): The study instrument had NOT been shown to have reliability or validity (if this was necessary). |
| 6. Was the same mode of data collection used for all subjects? | - Yes (LOW RISK): The same mode of data collection was used for all subjects. - No (HIGH RISK): The same mode of data collection was NOT used for all subjects. |
| 7. Was the length of the shortest prevalence period for the parameter of interest appropriate? | - Yes (LOW RISK): The shortest prevalence period for the parameter of interest was appropriate (e.g. point prevalence, one-week prevalence, one-year prevalence). - No (HIGH RISK): The shortest prevalence period for the parameter of interest was not appropriate (e.g. lifetime prevalence) |
| 8. Were the numerator(s) and denominator(s) for the parameter of interest appropriate? | - Yes (LOW RISK): The paper presented appropriate numerator(s) AND denominator(s) for the parameter of interest (e.g. the prevalence of fever as a symptom). - No (HIGH RISK): The paper did present numerator(s) AND denominator(s) for the parameter of interest but one or more of these were inappropriate. |
| **9. Summary item on the overall risk of study bias** | |
| - LOW RISK OF BIAS: Further research is very unlikely to change our confidence in the estimate - MODERATE RISK OF BIAS: Further research is likely to have an important impact on our confidence in the estimate and may change the estimate - HIGH RISK OF BIAS: Further research is very likely to have an important impact on our confidence in the estimate and is likely to change the estimate. | |
