# Supplementary material for: Clinical manifestations of Rift Valley fever in humans: Systematic review and meta-analysis
Source: PLoS Negl Trop Dis. 2022 Mar 25;16(3):e0010233. doi: 10.1371/journal.pntd.0010233 (PMC8986116; doi:10.1371/journal.pntd.0010233)
Supplement: S6 Table — (DOCX) [file pntd.0010233.s016.docx]

| **Author/ report year** | **Selection bias minimal** | **Non-response bias minimal** | **Data NOT collected from proxy** | **Case definition used** | **Reliability and validity assessed** | **Same mode of data collection used** | **Length prevalence period appropriate** | **Numerator and denominator appropriate** | **Summary of overall risk of bias** |
| --- | --- | --- | --- | --- | --- | --- | --- | --- | --- |
| El Imam; 2009 [1] | No | Yes | Yes | Yes | No | Yes | Yes | Yes | Moderate |
| Henderson; 1972 [2] | No | Yes | Yes | No | No | Yes | Yes | Yes | Moderate |
| Baudin; 2016 [3] | Yes | Yes | Yes | Yes | No | Yes | Yes | Yes | Low |
| Kahlon; 2010 [4] | No | Yes | Yes | Yes | No | Yes | Yes | No | Moderate |
| Laughlin; 1979 and Siam; 1980 [5, 6] | No | Yes | No | No | No | Yes | Yes | No | High |
| Mohammed Al-Hazmi; 2003 [7] | No | Yes | Yes | Yes | No | Yes | Yes | Yes | Moderate |
| Swanepoel; 1979 [8] | Yes | Yes | Yes | No | No | Yes | Yes | No | Moderate |
| Lagare; 2019 [9] | Yes | Yes | Yes | Yes | No | Yes | Yes | Yes | Low |
| Nguku; 2010 [10] | Yes | No | Yes | Yes | No | Yes | Yes | Yes | Moderate |
| Adam; 2010 [11] | No | Yes | Yes | Yes | No | Yes | Yes | Yes | Moderate |
| Ali Al-Hazmi; 2005 [12] | No | Yes | Yes | Yes | No | Yes | Yes | Yes | Low |
| Archer; 2011 [13] | Yes | Yes | Yes | Yes | No | Yes | Yes | Yes | Low |
| Kahiry; 2005 [14] | Yes | Yes | Yes | Yes | No | Yes | Yes | Yes | Low |
| Van Yelden; 1977 [15] | No | Yes | Yes | Yes | No | Yes | Yes | No | Moderate |
| Madani; 2003 [16] | No | Yes | Yes | Yes | No | Yes | Yes | Yes | Low |
| Joubert; 1951 [17] | Yes | Yes | Yes | No | No | Yes | Yes | No | Moderate |
| Sow and Faye; 2014 [18] | Yes | Yes | Yes | Yes | No | Yes | Yes | Yes | Low |
| Faye; 2007 [19] | No | No | No | Yes | No | No | Yes | No | High |
| St. Maurice; 2016 and 2018 [20, 21] | Yes | No | Yes | No | No | Yes | Yes | No | High |
| Gonzalez; 1987 [22] | No | No | Yes | No | No | No | No | No | High |
| Abdel-Wahab; 1978 [23] | No | No | Yes | No | No | No | Yes | No | High |
| Jouan; 1988 [24] | No | No | Yes | No | No | Yes | Yes | Yes | Moderate |
| Boushab; 2016 [25] | No | No | Yes | Yes | No | Yes | Yes | Yes | Moderate |
| Sow; 2016 [26] | Yes | No | Yes | Yes | No | Yes | Yes | Yes | Moderate |
| Anyangu; 2010 [27] | Yes | Yes | Yes | Yes | No | Yes | Yes | Yes | Low |
| Francis; 1935 [28] | No | Yes | Yes | No | No | Yes | Yes | No | Moderate |
| Kitchen; 1933 [29] | No | Yes | Yes | No | No | Yes | Yes | No | Moderate |
| Shrire; 1951 [30] | No | Yes | Yes | No | No | Yes | Yes | No | Moderate |
| Smithburn; 1949 [31] | No | Yes | Yes | No | No | Yes | Yes | No | Moderate |
| Mundel; 1951 [32] | No | Yes | Yes | No | No | Yes | Yes | No | Moderate |
| Legend: Low - Further research is very unlikely to change our confidence in the estimate; Moderate - Further research is likely to have an important impact on our confidence in the estimate and may change the estimate; High - Further research is very likely to have an important impact on our confidence in the estimate and is likely to change the estimate. | | | | | | | |  |  |

**S6 Table. Risk of bias in included studies for the clinical manifestations of Rift Valley fever (Yes = Low risk; No = High risk)**

References

1. El Imam MES, M.; Omran, M.; Abdalkareem, A.; El Gaili Mohamed, M. A.; Elbashir, A.; Khalafala, O. Acute renal failure associated with the Rift Valley fever: a single center study. Saudi Journal of Kidney Diseases & Transplantation. 2009;20(6):1047-52. PubMed PMID: 19861868.
2. Henderson BEM, A. W. R.; Kirya, B. G. Arbovirus epizootics involving man, mosquitoes and vertebrates at Lunyo, Uganda 1968. Annals of Tropical Medicine and Parasitology. 1972;66(3):343-55. PubMed PMID: 293047411.
3. Baudin M, Jumaa AM, Jomma HJ, Karsany MS, Bucht G, Näslund J, et al. Association of Rift Valley fever virus infection with miscarriage in Sudanese women: a cross-sectional study. The Lancet Global Health. 2016;4(11):e864-e71.
4. Kahlon SSP, C. J.; LeDuc, J.; Muchiri, E. M.; Muiruri, S.; Njenga, M. K.; Breiman, R. F.; White Jr, A. C.; King, C. H. Case report: Severe rift valley fever may present with a characteristic clinical syndrome. American Journal of Tropical Medicine and Hygiene. 2010;82(3):371-5. doi: <http://dx.doi.org/10.4269/ajtmh.2010.09-0669>. PubMed PMID: 358507949.
5. Laughlin LWM, J. M.; Strausbaugh, L. J.; Morens, D. M.; Watten, R. H. Epidemic Rift Valley fever in Egypt: observations of the spectrum of human illness. Transactions of the Royal Society of Tropical Medicine and Hygiene. 1979;73(6):630-3.
6. Siam A, Meegan J, Gharbawi K. Rift Valley fever ocular manifestations: observations during the 1977 epidemic in Egypt. Br J Ophthalmol. 1980;64(5):366-74.
7. Al-Hazmi M, Ayoola EA, Abdurahman M, Banzal S, Ashraf J, El-Bushra A, et al. Epidemic Rift Valley fever in Saudi Arabia: a clinical study of severe illness in humans. Clinical infectious diseases. 2003;36(3):245-52.
8. Swanepoel RM, B.; Watt, J. A. Fatal Rift Valley fever of man in Rhodesia. Central African Journal of Medicine. 1979;25(1):1-8. PubMed PMID: 421262.
9. Lagare AF, G.; Ibrahim, A.; Ousmane, S.; Sadio, B.; Abdoulaye, M.; Alhassane, A.; Mahaman, A. E.; Issaka, B.; Sidikou, F.; Zaneidou, M.; Bienvenue, B.; Mamoudou, H. D.; Diallo, A. B.; Kadade, G.; Testa, J.; Mainassara, H. B.; Faye, O. First occurrence of Rift Valley fever outbreak in Niger, 2016. Veterinary Medicine and Science. 2019;5(1):70-8. doi: 10.1002/vms3.135. PubMed PMID: WOS:000458894500008.
10. Nguku PM, Sharif S, Mutonga D, Amwayi S, Omolo J, Mohammed O, et al. An investigation of a major outbreak of Rift Valley fever in Kenya: 2006–2007. The American journal of tropical medicine and hygiene. 2010;83(2_Suppl):05-13.
11. Adam AAK, M. S.; Adam, I. Manifestations of severe Rift Valley fever in Sudan: International Journal of Infectious Diseases; 2010. 14(2):e179-e180.; 2010.
12. Al-Hazmi A, Al-Rajhi AA, Abboud EB, Ayoola EA, Al-Hazmi M, Saadi R, et al. Ocular complications of Rift Valley fever outbreak in Saudi Arabia. Ophthalmology. 2005;112(2):313-8.
13. Archer BNW, J.; Paweska, J.; Nkosi, D.; Leman, P.; Tint, K. S.; Blumberg, L. Outbreak of Rift Valley fever affecting veterinarians and farmers in South Africa, 2008. South African Medical Journal Suid-Afrikaanse Tydskrif Vir Geneeskunde. 2011;101(4):263-6. PubMed PMID: 21786732.
14. Kahiry W. Pattern of positive Rift Valley Fever (RVF) cases during the epidemic period Sep.-Dec. 2000 in Al-Zuhrah District-Hodiedah Governorate- Yemen. University of Aden Journal of Natural and Applied Sciences. 2005;9(3):597-607.
15. Van Velden DJJM, J. D.; Olivier, J. Rift Valley fever affecting humans in South Africa. A clinicopathological study. South African Medical Journal. 1977;51(24):867-71. PubMed PMID: 8135309.
16. Madani TA, Al-Mazrou YY, Al-Jeffri MH, Mishkhas AA, Al-Rabeah AM, Turkistani AM, et al. Rift Valley fever epidemic in Saudi Arabia: epidemiological, clinical, and laboratory characteristics. Clinical Infectious Diseases. 2003;37(8):1084-92.
17. Joubert JDSF, A. L.; Gear, J. Rift Valley Fever in South Africa. 2. The Occurrence of Human Cases in the Orange Free State, the North-western Cape Province, the Western and Southern Transvaal. A. Epidemlologlcal and Clinical Findings. [not specified]. South African Medical Journal. 1951;25(48):890-91.
18. Sow AB, Y.; Ba, H.; Diallo, D.; Faye, O.; Loucoubar, C.; Boushab, M.; Barry, Y.; Diallo, M.; Sall, A. A. Rift valley fever outbreak, Southern Mauritania, 2012. Emerging Infectious Diseases. 2014;20(2):296-9. doi: <http://dx.doi.org/10.3201/eid2002.131000>. PubMed PMID: 372137240.
19. Faye OD, M.; Diop, D.; Bezeid, O. E.; Ba, H.; Niang, M.; Dia, I.; Mohamed, S. A. O.; Ndiaye, K.; Diallo, D.; Ly, P. O.; Diallo, B.; Nabeth, P.; Simon, F.; Lo, B.; Diop, O. M. Rift valley fever outbreak with East-Central African virus lineage in Mauritania, 2003. Emerging Infectious Diseases. 2007;13(7):1016-23.
20. St. Maurice AdN, L.; Purpura, L.; Ervin, E.; Tumusiime, A.; Balinandi, S.; Kayondo, J.; Mulei, S.; Namutebi, A. M.; Tusiime, P.; Wiersma, S.; Nichol, S.; Rollin, P.; Klena, J.; Knust, B.; Shoemaker, T. Rift Valley fever response - Kabale District, Uganda, March 2016. Morbidity and Mortality Weekly Report. 2016;65(43):1200-1. doi: <http://dx.doi.org/10.15585/mmwr.mm6543a5>.
21. St. Maurice AdH, J.; Nyakarahuka, L.; Balinandi, S.; Tumusiime, A.; Kyondo, J.; Mulei, S.; Namutebi, A.; Knust, B.; Shoemaker, T.; Nichol, S. T.; McElroy, A. K.; Spiropoulou, C. F. Rift Valley fever viral load correlates with the human inflammatory response and coagulation pathway abnormalities in humans with hemorrhagic manifestations. PLoS Neglected Tropical Diseases. 2018;12(5).
22. Gonzalez JPB, J. C.; Lesbordes, J. L.; Madelon, M. C.; Mathiot, C. C.; Meunier, D. M. Y.; Georges, A. J. Rift Valley fever virus and haemorrhagic fever in the Central African Republic. Annales de l'Institut Pasteur Virology. 1987;138(3):385-90. PubMed PMID: 17140282.
23. Abdel-Wahab KSEDEB, L. M.; El-Tayeb, E. M.; Omar, H.; Ossman, M. A. M.; Yasin, W. Rift Valley Fever virus infections in Egypt: pathological and virological findings in man. Transactions of the Royal Society of Tropical Medicine and Hygiene. 1978;72(4):392-6.
24. Jouan AG, B. le; Digoutte, J. P.; Philippe, B.; Riou, O.; Adam, F. An RVF epidemic in southern Mauritania. Annales de l'Institut Pasteur, Virology. 1988;139(3):307-8.
25. Boushab BM, Fall-Malick FZ, Ould Baba SEW, Ould Salem ML, Belizaire MRD, Ledib H, et al. Severe Human Illness Caused by Rift Valley Fever Virus in Mauritania, 2015. Open forum infectious diseases. 2016;3(4):ofw200-ofw. doi: 10.1093/ofid/ofw200. PubMed PMID: 27844026.
26. Sow AB, Y.; Diallo, D.; Fall, G.; Faye, O.; Bob, N. S.; Loucoubar, C.; Richard, V.; Dia, A. T.; Diallo, M.; Malvy, D.; Sall, A. A. Widespread Rift Valley Fever Emergence in Senegal in 2013-2014. Open Forum Infectious Diseases. 2016;3 (3) (no pagination)(ofw149). doi: <http://dx.doi.org/10.1093/ofid/ofw149>. PubMed PMID: 614929832.
27. Anyangu ASG, L. H.; Sharif, S. K.; Nguku, P. M.; Omolo, J. O.; Mutonga, D.; Rao, C. Y.; Lederman, E. R.; Schnabel, D.; Paweska, J. T.; Katz, M.; Hightower, A.; Njenga, M. K.; Feikin, D. R.; Breiman, R. F. Risk factors for severe Rift Valley fever infection in Kenya, 2007. American Journal of Tropical Medicine & Hygiene. 2010;83(2 Suppl):14-21. doi: <https://dx.doi.org/10.4269/ajtmh.2010.09-0293>. PubMed PMID: 20682901.
28. Francis T, Magill T. Rift Valley fever: a report of three cases of laboratory infection and the experimental transmission of the disease to ferrets. Journal of Experimental Medicine. 1935;62(3):433-48.
29. Kitchen S. Laboratory Infections with the Virus of Rift Valley Fever. The American Journal of Tropical Medicine and Hygiene. 1934;1(6):547-64.
30. Shirire. Macular changes in Rift Valley fever. South African Medical Journal. 1951;25(50):926-30.
31. Smithburn K, Mahaffy A, Haddow A, Kitchen S, Smith J. Rift Valley fever: accidental infections among laboratory workers. The Journal of Immunology. 1949;62(2):213-27.
32. Gear MBJ. Rift Valley fever the occurrence of human cases in Johannesburg. S Afr Med J. 1951;25(44):797-800. PubMed Central PMCID: PMCPMID: 14892917.
