# Supplementary material for: Clinical manifestations of Rift Valley fever in humans: Systematic review and meta-analysis
Source: PLoS Negl Trop Dis. 2022 Mar 25;16(3):e0010233. doi: 10.1371/journal.pntd.0010233 (PMC8986116; doi:10.1371/journal.pntd.0010233)
Supplement: S7 Table — (DOCX) [file pntd.0010233.s017.docx]

**S7 Table. Rift Valley fever case definitions use and clinical syndromes manifested by patients in the respective outbreaks**

| **Outbreak dates and country** | **Studies/papers** | **Case definition and syndromes covered by case definition** | | **Syndromes manifested by patients in the outbreak** |
| --- | --- | --- | --- | --- |
|  |  | **Case definition used** | **Syndromes covered by case definition** |  |
| Sep - Nov 2007  Sudan | Adam 2010 [1] | No |  | Hepatic, Renal, Neurological, Hemorrhagic, Visual, Other - death |
| Sep 2007 - Jan 2008  Sudan | El Imam 2009 [2] | No |  | Febrile, Gastro-enteritis, Hepatic, Neurological, Haemorrhagic, Visual, Rena, Other - death |
| Jun 2011 – Nov 2012  Sudan | Baudin 2016 [3] | Yes | Haemorrhagic | Gastroenteritis, Haemorrhagic, Obstetric |
| Dec 1944 – Apr 1948  Uganda | Smithburn 1949 [4] | No |  | Febrile, Neurological |
| April - May 1968  Uganda | Henderson 1972 [5] | No |  | Febrile |
| Mar – Jun 2016  Uganda | St. Maurice 2016 [6]  St. Maurice 2018 [7] | No |  | Febrile, Gastroenteritis, Hepatic, Haemorrhagic |
| Nov 2006 - Mar 2007  Kenya | Nguku 2010 [8] | Yes | Febrile, Haemorrhagic | Febrile, Hepatic, Haemorrhagic, Visual |
| Dec 2006 - Feb 2007  Kenya | Kahlon 2010 [9] | Yes | Febrile, Neurological, Haemorrhagic, Visual | Febrile, Hepatic, Neurological, Haemorrhagic, Visual, Other - death |
| Jan - Mar 2007  Kenya | Anyangu 2010 [10] | Yes | Febrile, Haemorrhagic | Haemorrhagic, Neurological, Haemorrhagic, Other - death |
| Oct – Dec 1977  Egypt | Abdel-Wahab 1978 [11] | No |  | Febrile, Hepatic, Neurological, Haemorrhagic, Other - death |
| Oct - Dec 1977  Egypt | Laughlin 1979 [12]  Siam 1980 [13] | No |  | Febrile, Hepatic, Neurological, Haemorrhagic, Visual, Other - death |
| Aug 2000 - Sep 2001  Saudi Arabia | Madani 2003 [14] | Yes | Febrile, Gastroenteritis, Hepatic, Neurological, Haemorrhagic, Visual, Other – unexplained death | Febrile, Gastroenteritis, Hepatic, Renal, Neurological, Haemorrhagic, Visual, Other - death |
| Sep - Nov 2000  Saudi Arabia | Mohammed Al-Hazmi 2003 [15] | Yes | Febrile, Gastroenteritis, Hepatic, Neurological, Haemorrhagic, Visual, Other – unexplained death | Febrile, Gastroenteritis, Hepatic, Renal, Neurological, Haemorrhagic, Visual, Other - death |
| Sep – Nov 2000  Saudi Arabia | Ali Al-Hazmi 2005 [16] | Yes | Febrile, Gastroenteritis, Hepatic, Neurological, Haemorrhagic, Visual, Other – unexplained death | Hepatic, Renal, Neurological, Hemorrhagic, Visual |
| Sep – Dec 2000  Yemen | Kahiry 2005 [17] | Yes | Febrile, Hepatic, Neurological, Haemorrhagic, Visual, Other – unexplained death | Febrile, Gastroenteritis, Hepatic, Neurological, Haemorrhagic, Visual, Other - death |
| Feb – Jun 1977  Zimbabwe | Swanepoel 1979 [18] | No |  | Febrile, Gastroenteritis, Hepatic, Neurological, Haemorrhagic, Visual, Cardiopulmonary, Other - death |
| Aug - Dec 2016  Niger | Lagare 2019 [19] | No |  | Febrile, Hepatic, Haemorrhagic, Other - death |
| Mar - May 1951  South Africa | Joubert 1951 [20] | No |  | Febrile, Neurological, Visual |
| Mar - Jun 1951  South Africa | Shrire 1951 [21] | No |  | Febrile, Visual |
| April 1951  South Africa | Mundel 1951 [22] | No |  | Febrile, Neurological, Haemorrhagic |
| Mar - May 1975  South Africa | Van Velden 1977 [23] | Yes |  | Febrile/Classic, Gastroenteritis, Neurological, Haemorrhagic, Other - death |
| Feb – Mar 2008  South Africa | Archer 2011 [24] | Yes | Febrile, Hepatic, Neurological, Haemorrhagic, Visual | Febrile, Neurological |
| Oct 1987  Mauritania | Jouan 1988 [25] | No |  | Febrile, Hepatic, Neurological, Haemorrhagic, Other - death |
| Sep – Dec 2003  Mauritania | Faye 2007 [26] | Yes | Febrile, Hepatic, Neurological, Haemorrhagic, Other – unexplained death | Febrile, Hepatic, Haemorrhagic |
| Sep - Nov 2012  Mauritania | Sow & Faye 2014 [27] | Yes | Febrile, Neurological, Haemorrhagic, Other – unexplained death | Febrile, Haemorrhagic, Other - death |
| Sep - Nov 2015  Mauritania | Boushab 2016 [28] | Yes | Febrile, Neurological, Haemorrhagic, Visual | Febrile, Hepatic, Neurological, Hemorrhagic, Other - death |
| 1971 – 1986  Central African Republic | Gonzalez 1987 [29] | No |  | Febrile, Renal, Haemorrhagic, Other - death |
| Sep 2013 – Feb 2014  Senegal | Sow 2016 [30] | Yes | Febrile, Hepatic, Neurological, Haemorrhagic, Visual | Febrile, Hepatic, Neurological, Visual |
| Feb - Oct 1933  USA | Kitchen 1933 [31] | No |  | Febrile, Gastroenteritis, Neurological |
| Oct – Dec 1934  USA | Francis 1935 [32] | No |  | Febrile |
| **Total:**  **Outbreaks = 21**  **Countries = 13** | **Number of studies = 30 (32 reports)** | **Case definition used in 14/30 studies** | **Case definitions captured 1 – 6 syndromes,** | **Outbreaks revealed 1-9 syndromes** |

**References**

1. Adam AAK, M. S.; Adam, I. Manifestations of severe Rift Valley fever in Sudan: International Journal of Infectious Diseases; 2010. 14(2):e179-e180.; 2010.
2. El Imam MES, M.; Omran, M.; Abdalkareem, A.; El Gaili Mohamed, M. A.; Elbashir, A.; Khalafala, O. Acute renal failure associated with the Rift Valley fever: a single center study. Saudi Journal of Kidney Diseases & Transplantation. 2009;20(6):1047-52. PubMed PMID: 19861868.
3. Baudin M, Jumaa AM, Jomma HJ, Karsany MS, Bucht G, Näslund J, et al. Association of Rift Valley fever virus infection with miscarriage in Sudanese women: a cross-sectional study. The Lancet Global Health. 2016;4(11):e864-e71.
4. Smithburn K, Mahaffy A, Haddow A, Kitchen S, Smith J. Rift Valley fever: accidental infections among laboratory workers. The Journal of Immunology. 1949;62(2):213-27.
5. Henderson BEM, A. W. R.; Kirya, B. G. Arbovirus epizootics involving man, mosquitoes and vertebrates at Lunyo, Uganda 1968. Annals of Tropical Medicine and Parasitology. 1972;66(3):343-55. PubMed PMID: 293047411.
6. St. Maurice AdN, L.; Purpura, L.; Ervin, E.; Tumusiime, A.; Balinandi, S.; Kayondo, J.; Mulei, S.; Namutebi, A. M.; Tusiime, P.; Wiersma, S.; Nichol, S.; Rollin, P.; Klena, J.; Knust, B.; Shoemaker, T. Rift Valley fever response - Kabale District, Uganda, March 2016. Morbidity and Mortality Weekly Report. 2016;65(43):1200-1. doi: <http://dx.doi.org/10.15585/mmwr.mm6543a5>.
7. St. Maurice AdH, J.; Nyakarahuka, L.; Balinandi, S.; Tumusiime, A.; Kyondo, J.; Mulei, S.; Namutebi, A.; Knust, B.; Shoemaker, T.; Nichol, S. T.; McElroy, A. K.; Spiropoulou, C. F. Rift Valley fever viral load correlates with the human inflammatory response and coagulation pathway abnormalities in humans with hemorrhagic manifestations. PLoS Neglected Tropical Diseases. 2018;12(5).
8. Nguku PM, Sharif S, Mutonga D, Amwayi S, Omolo J, Mohammed O, et al. An investigation of a major outbreak of Rift Valley fever in Kenya: 2006–2007. The American journal of tropical medicine and hygiene. 2010;83(2_Suppl):05-13.
9. Kahlon SSP, C. J.; LeDuc, J.; Muchiri, E. M.; Muiruri, S.; Njenga, M. K.; Breiman, R. F.; White Jr, A. C.; King, C. H. Case report: Severe rift valley fever may present with a characteristic clinical syndrome. American Journal of Tropical Medicine and Hygiene. 2010;82(3):371-5. doi: <http://dx.doi.org/10.4269/ajtmh.2010.09-0669>. PubMed PMID: 358507949.
10. Anyangu ASG, L. H.; Sharif, S. K.; Nguku, P. M.; Omolo, J. O.; Mutonga, D.; Rao, C. Y.; Lederman, E. R.; Schnabel, D.; Paweska, J. T.; Katz, M.; Hightower, A.; Njenga, M. K.; Feikin, D. R.; Breiman, R. F. Risk factors for severe Rift Valley fever infection in Kenya, 2007. American Journal of Tropical Medicine & Hygiene. 2010;83(2 Suppl):14-21. doi: <https://dx.doi.org/10.4269/ajtmh.2010.09-0293>. PubMed PMID: 20682901.
11. Abdel-Wahab KSEDEB, L. M.; El-Tayeb, E. M.; Omar, H.; Ossman, M. A. M.; Yasin, W. Rift Valley Fever virus infections in Egypt: pathological and virological findings in man. Transactions of the Royal Society of Tropical Medicine and Hygiene. 1978;72(4):392-6.
12. Laughlin LWM, J. M.; Strausbaugh, L. J.; Morens, D. M.; Watten, R. H. Epidemic Rift Valley fever in Egypt: observations of the spectrum of human illness. Transactions of the Royal Society of Tropical Medicine and Hygiene. 1979;73(6):630-3.
13. Siam A, Meegan J, Gharbawi K. Rift Valley fever ocular manifestations: observations during the 1977 epidemic in Egypt. Br J Ophthalmol. 1980;64(5):366-74.
14. Madani TA, Al-Mazrou YY, Al-Jeffri MH, Mishkhas AA, Al-Rabeah AM, Turkistani AM, et al. Rift Valley fever epidemic in Saudi Arabia: epidemiological, clinical, and laboratory characteristics. Clinical Infectious Diseases. 2003;37(8):1084-92.
15. Al-Hazmi M, Ayoola EA, Abdurahman M, Banzal S, Ashraf J, El-Bushra A, et al. Epidemic Rift Valley fever in Saudi Arabia: a clinical study of severe illness in humans. Clinical infectious diseases. 2003;36(3):245-52.
16. Al-Hazmi A, Al-Rajhi AA, Abboud EB, Ayoola EA, Al-Hazmi M, Saadi R, et al. Ocular complications of Rift Valley fever outbreak in Saudi Arabia. Ophthalmology. 2005;112(2):313-8.
17. Kahiry W. Pattern of positive Rift Valley Fever (RVF) cases during the epidemic period Sep.-Dec. 2000 in Al-Zuhrah District-Hodiedah Governorate- Yemen. University of Aden Journal of Natural and Applied Sciences. 2005;9(3):597-607.
18. Swanepoel RM, B.; Watt, J. A. Fatal Rift Valley fever of man in Rhodesia. Central African Journal of Medicine. 1979;25(1):1-8. PubMed PMID: 421262.
19. Lagare AF, G.; Ibrahim, A.; Ousmane, S.; Sadio, B.; Abdoulaye, M.; Alhassane, A.; Mahaman, A. E.; Issaka, B.; Sidikou, F.; Zaneidou, M.; Bienvenue, B.; Mamoudou, H. D.; Diallo, A. B.; Kadade, G.; Testa, J.; Mainassara, H. B.; Faye, O. First occurrence of Rift Valley fever outbreak in Niger, 2016. Veterinary Medicine and Science. 2019;5(1):70-8. doi: 10.1002/vms3.135. PubMed PMID: WOS:000458894500008.
20. Joubert JDSF, A. L.; Gear, J. Rift Valley Fever in South Africa. 2. The Occurrence of Human Cases in the Orange Free State, the North-western Cape Province, the Western and Southern Transvaal. A. Epidemlologlcal and Clinical Findings. [not specified]. South African Medical Journal. 1951;25(48):890-91.
21. Shirire. Macular changes in Rift Valley fever. 1951.
22. Gear MBJ. Rift Valley fever the occurrence of human cases in Johannesburg. 1951.
23. Van Velden DJJM, J. D.; Olivier, J. Rift Valley fever affecting humans in South Africa. A clinicopathological study. South African Medical Journal. 1977;51(24):867-71. PubMed PMID: 8135309.
24. Archer BNW, J.; Paweska, J.; Nkosi, D.; Leman, P.; Tint, K. S.; Blumberg, L. Outbreak of Rift Valley fever affecting veterinarians and farmers in South Africa, 2008. South African Medical Journal Suid-Afrikaanse Tydskrif Vir Geneeskunde. 2011;101(4):263-6. PubMed PMID: 21786732.
25. Jouan AG, B. le; Digoutte, J. P.; Philippe, B.; Riou, O.; Adam, F. An RVF epidemic in southern Mauritania. Annales de l'Institut Pasteur, Virology. 1988;139(3):307-8.
26. Faye OD, M.; Diop, D.; Bezeid, O. E.; Ba, H.; Niang, M.; Dia, I.; Mohamed, S. A. O.; Ndiaye, K.; Diallo, D.; Ly, P. O.; Diallo, B.; Nabeth, P.; Simon, F.; Lo, B.; Diop, O. M. Rift valley fever outbreak with East-Central African virus lineage in Mauritania, 2003. Emerging Infectious Diseases. 2007;13(7):1016-23.
27. Sow AB, Y.; Ba, H.; Diallo, D.; Faye, O.; Loucoubar, C.; Boushab, M.; Barry, Y.; Diallo, M.; Sall, A. A. Rift valley fever outbreak, Southern Mauritania, 2012. Emerging Infectious Diseases. 2014;20(2):296-9. doi: <http://dx.doi.org/10.3201/eid2002.131000>. PubMed PMID: 372137240.
28. Boushab BM, Fall-Malick FZ, Ould Baba SEW, Ould Salem ML, Belizaire MRD, Ledib H, et al. Severe Human Illness Caused by Rift Valley Fever Virus in Mauritania, 2015. Open forum infectious diseases. 2016;3(4):ofw200-ofw. doi: 10.1093/ofid/ofw200. PubMed PMID: 27844026.
29. Gonzalez JPB, J. C.; Lesbordes, J. L.; Madelon, M. C.; Mathiot, C. C.; Meunier, D. M. Y.; Georges, A. J. Rift Valley fever virus and haemorrhagic fever in the Central African Republic. Annales de l'Institut Pasteur Virology. 1987;138(3):385-90. PubMed PMID: 17140282.
30. Sow AB, Y.; Diallo, D.; Fall, G.; Faye, O.; Bob, N. S.; Loucoubar, C.; Richard, V.; Dia, A. T.; Diallo, M.; Malvy, D.; Sall, A. A. Widespread Rift Valley Fever Emergence in Senegal in 2013-2014. Open Forum Infectious Diseases. 2016;3 (3) (no pagination)(ofw149). doi: <http://dx.doi.org/10.1093/ofid/ofw149>. PubMed PMID: 614929832.
31. Kitchen SF. Laboratory infections with the virus of Rift Valley fever. 1934.
32. Francis T, Magill T. Rift Valley fever: a report of three cases of laboratory infection and the experimental transmission of the disease to ferrets. Journal of Experimental Medicine. 1935;62(3):433-48.
