# Supplementary material for: Clinical manifestations of Rift Valley fever in humans: Systematic review and meta-analysis
Source: PLoS Negl Trop Dis. 2022 Mar 25;16(3):e0010233. doi: 10.1371/journal.pntd.0010233 (PMC8986116; doi:10.1371/journal.pntd.0010233)
Supplement: S8 Table — (DOCX) [file pntd.0010233.s018.docx]

**S8 Table. Characteristics of RVF clinical symptoms in humans**

| **Symptom** | **Summary of symptom description** | **Number of studies describing symptom out of studies that reported on symptom** | **Number of studies description of symptom not reported** |
| --- | --- | --- | --- |
| **General febrile syndrome** | | | |
| **Fever** | Sudden onset [1-4]; mild fever [5] or very high fever [1-4]; temperature >39⁰C [1, 2]; recurring fever [1]; biphasic pattern / saddleback rise in temperature [6, 7]; fever for 2-4 days, free for 1-2 days, relapse for 1-3days, then recovery [3, 6, 8] | 11/22 | 5 |
| **Headache** | Sudden/acute onset [1-4, 6, 9]; mild [5]; severe [1, 3, 6, 10]; throbbing [11]; diffuse [11] or frontal [4, 11, 12]; associated retro-orbital pain [2] | 10/21 |  |
| **Arthralgia** | Sudden onset [3, 4, 6, 9]; slow onset [6]; no arthralgia [6, 11]; mild [5]; severe [3, 10, 13]; varied severity (mainly proximal large joints: knee, elbow, shoulder), no tenderness, effusion or pain on active and passive joint movement [11]; excruciating pain in knees [3]; associated stiffness in all joints [6] | 8/16 |  |
| **Myalgia** | Sudden onset [1, 4, 6, 9]; slow onset [6]; no myalgia [6]; mild [5]; backbreaking myalgia [1]; shooting myalgias [14]; muscular weakness persists longer than fever [12] | 7/15 |  |
| **Backache** | Sudden onset [9]; slow onset [6]; no backache [6]; severe [6] | 2/6 |  |
| **Chills** | Sudden onset [6, 9]; severe [14] | 3/9 |  |
| **Rigor** | Sudden onset [2, 6] | 2/5 |  |
| **Malaise** | Sudden onset [1, 6]; severe [13] | 3/8 |  |
| **Fatigue** | Easy fatiguegability on exertion [12] | 1/3 |  |
| **Weight loss** | Subjective weight loss within first few days [11] | 1/1 |  |
| **Gastrointestinal syndrome** | | | |
| **Epigastric discomfort** | Vague mid-epigastric discomfort, occurs early during the illness [11] | 1/2 | 10 |
| **Abdominal pain** | Poorly localised abdominal pain [15]; tenderness in right upper quadrant of abdomen, no palpable liver or spleen [2] | 2/11 |  |
| **Anorexia** | Persistent anorexia (continues for a long time during recovery)[11]; anorexia and definitive aversion to food; onset (71% sudden, 29% slow) [6] | 2/8 |  |
| **Nausea/vomiting** | Sudden onset [2] | 1/16 |  |
| **Hepatic syndrome** | |  |  |
| **Jaundice** | Jaundice occurs within 2 to 21 days [1, 11] | 2/15 |  |
| **Hepatomegaly** | Mild [1]; tender and palpable hepatomegaly [2, 11] | 3/5 |  |
| **Right hypochondriac tenderness** | Pain over liver, tender on pressure [3] | 1/4 |  |
| **Splenomegaly** | Mild [1, 2] | 2/5 |  |
| **Renal syndrome** | | | |
| **Renal failure** | Oliguria; pedal and/or pulmonary oedema; renal failure entirely acute without progression to chronic renal failure [16] | 1/3 |  |
| **Neurological/Meningoencephalitis syndrome** | | | |
| **Central nervous system symptoms/encephalitis** | All patients with meningoencephalitis had a cerebrospinal fluid (CSF) pleocytosis but normal CSF glucose and protein concentrations. The CSF cell counts ranged from 20 to 600 cells/mm³, predominantly lymphocytes [1] and numb feeling along the spine and legs [6] | 2/8 | **26** |
| **Delirium** | Delirium coinciding with peak of clinical severity or onset of haemorrhage [11] | 1/4 |  |
| **Haemorrhagic syndrome** | | | |
| **Haemorrhagic disease (bleeding from any site)** | Onset within 2-4days. Severe coagulopathy with marked prolongation of prothrombin time and partial thromboplastin time which may present as disseminated intravascular coagulopathy [15], with severe /profuse bleeding [9] from multiple sites including the nose, gums, vomit and skin rashes [11]. Gastrointestinal bleeding may present as frank blood in stool [11] or old blood with the appearance of coffee grounds [2]. Marked bleeding may lead to anaemia [9]. | 6/16 | 14 |
| **Macular/Purpura rash** | Multiple macular and purpuric rashes [1, 2] | 2/3 |  |
| **Petechiae** | Multiple petechiae [1, 17] | 1/5 |  |
| **Epistaxis** | Mild but persistent nose bleeding [14] | 1/10 |  |
| **Visual/ocular syndrome** | | | |
| **Visual disturbance** | Diminished visual acuity varied from light perception to finger counting develop within 2-7 days and persist for 10 - 15 days after onset of illness [1]. Severe visual impairment develop after 4 weeks following onset of illness and presents as blurred vision, fogginess or haze, floating black spots or gap within visual fields [8, 11]. Fundoscopy: Macular, paramacular or extra-macular exudates [1, 17]. May appear as multiple yellow plaques of variable sizes [8, 11] with mild erythema at the borders [11] and associated haemorrhage [1, 8, 17] or retinal detachment [8]. Severe uveitis with keratic precipitates and vitreous haze, vasculitis with peripapillary choroidal ischaemia and infarction, severe cases vessels may be sheathed, occluded, and optic atrophy may occur [1, 17]. Symptoms largely resolve within 2 weeks [11] but may persist for 3 months after acute onset [3] | 4/12 | 7 |
| **Eye pain** | Sudden onset [9]; pain behind the eyeball [12] | 2/3 |  |
| **Cardio-respiratory syndrome** | | | |
| **Throat swelling** | Throat reddened [2] | 1/2 | 4 |
| **Cough** | Slight cough [14] | 1/4 |  |
| **Cardiovascular / Myocarditis** | Hypotension without tachycardia, systolic BP 60-70mmHg [11]; tightness over chest as sign of coronary thrombosis | 2/3 |  |
| **Obstetric syndrome** | |  |  |
| **Abortion or miscarriage** | RVF increase risk of miscarriage by 7 times and patients experience severe haemorrhage [13] | 1/1 |  |
| **Death** | | | |
| **Death** | Isolated acute hepatic or renal failure or hepatorenal impairment is each associated with 21-40% mortality occurring within the first week of illness [1, 16-18] | 3/16 | 13 |

**References**

1. Laughlin LWM, J. M.; Strausbaugh, L. J.; Morens, D. M.; Watten, R. H. Epidemic Rift Valley fever in Egypt: observations of the spectrum of human illness. Transactions of the Royal Society of Tropical Medicine and Hygiene. 1979;73(6):630-3.
2. Swanepoel RM, B.; Watt, J. A. Fatal Rift Valley fever of man in Rhodesia. Central African Journal of Medicine. 1979;25(1):1-8. PubMed PMID: 421262.
3. Joubert JDSF, A. L.; Gear, J. Rift Valley Fever in South Africa. 2. The Occurrence of Human Cases in the Orange Free State, the North-western Cape Province, the Western and Southern Transvaal. A. Epidemlologlcal and Clinical Findings. [not specified]. South African Medical Journal. 1951;25(48):890-91.
4. Abdel-Wahab KSEDEB, L. M.; El-Tayeb, E. M.; Omar, H.; Ossman, M. A. M.; Yasin, W. Rift Valley Fever virus infections in Egypt: pathological and virological findings in man. Transactions of the Royal Society of Tropical Medicine and Hygiene. 1978;72(4):392-6.
5. Sow AB, Y.; Ba, H.; Diallo, D.; Faye, O.; Loucoubar, C.; Boushab, M.; Barry, Y.; Diallo, M.; Sall, A. A. Rift valley fever outbreak, Southern Mauritania, 2012. Emerging Infectious Diseases. 2014;20(2):296-9. doi: <http://dx.doi.org/10.3201/eid2002.131000>. PubMed PMID: 372137240.
6. Smithburn K, Mahaffy A, Haddow A, Kitchen S, Smith J. Rift Valley fever: accidental infections among laboratory workers. The Journal of Immunology. 1949;62(2):213-27.
7. Gear MBJ. Rift Valley fever the occurrence of human cases in Johannesburg. 1951.
8. Shirire. Macular changes in Rift Valley fever. 1951.
9. Van Velden DJJM, J. D.; Olivier, J. Rift Valley fever affecting humans in South Africa. A clinicopathological study. South African Medical Journal. 1977;51(24):867-71. PubMed PMID: 8135309.
10. Henderson BEM, A. W. R.; Kirya, B. G. Arbovirus epizootics involving man, mosquitoes and vertebrates at Lunyo, Uganda 1968. Annals of Tropical Medicine and Parasitology. 1972;66(3):343-55. PubMed PMID: 293047411.
11. Kahlon SSP, C. J.; LeDuc, J.; Muchiri, E. M.; Muiruri, S.; Njenga, M. K.; Breiman, R. F.; White Jr, A. C.; King, C. H. Case report: Severe rift valley fever may present with a characteristic clinical syndrome. American Journal of Tropical Medicine and Hygiene. 2010;82(3):371-5. doi: <http://dx.doi.org/10.4269/ajtmh.2010.09-0669>. PubMed PMID: 358507949.
12. Kitchen SF. Laboratory infections with the virus of Rift Valley fever. 1934.
13. Baudin M, Jumaa AM, Jomma HJ, Karsany MS, Bucht G, Näslund J, et al. Association of Rift Valley fever virus infection with miscarriage in Sudanese women: a cross-sectional study. The Lancet Global Health. 2016;4(11):e864-e71.
14. Francis T, Magill T. Rift Valley fever: a report of three cases of laboratory infection and the experimental transmission of the disease to ferrets. Journal of Experimental Medicine. 1935;62(3):433-48.
15. Al-Hazmi M, Ayoola EA, Abdurahman M, Banzal S, Ashraf J, El-Bushra A, et al. Epidemic Rift Valley fever in Saudi Arabia: a clinical study of severe illness in humans. Clinical infectious diseases. 2003;36(3):245-52.
16. El Imam MES, M.; Omran, M.; Abdalkareem, A.; El Gaili Mohamed, M. A.; Elbashir, A.; Khalafala, O. Acute renal failure associated with the Rift Valley fever: a single center study. Saudi Journal of Kidney Diseases & Transplantation. 2009;20(6):1047-52. PubMed PMID: 19861868.
17. Siam A, Meegan J, Gharbawi K. Rift Valley fever ocular manifestations: observations during the 1977 epidemic in Egypt. Br J Ophthalmol. 1980;64(5):366-74.
18. Adam AAK, M. S.; Adam, I. Manifestations of severe Rift Valley fever in Sudan: International Journal of Infectious Diseases; 2010. 14(2):e179-e180.; 2010.
