# Supplementary material for: Clinical manifestations of Rift Valley fever in humans: Systematic review and meta-analysis
Source: PLoS Negl Trop Dis. 2022 Mar 25;16(3):e0010233. doi: 10.1371/journal.pntd.0010233 (PMC8986116; doi:10.1371/journal.pntd.0010233)
Supplement: S9 Table — (DOCX) [file pntd.0010233.s019.docx]

**S9 Table. Proportion of patients with other RVF clinical manifestations for which a pooled prevalence could not be estimated**

| **Symptom** | **Number enrolled (N)** | **Number with symptom (n)** | **Non-pooled proportion with symptom (%)** | **Study** |
| --- | --- | --- | --- | --- |
| **General febrile/flu-like syndrome** | | | | |
| Weight loss | 6 | 4 | 67 | Kahlon 2010 [1] |
| Prostration | 8 | 3 | 38 | Smithburn 1949 [2] |
| Nasal congestion | 3 | 1 | 33 | Francis 1935 [3] |
| Dehydration | 165 | 41 | 24.8 | Mohammed A 2003 [4] |
| Joint stiffness | 8 | 1 | 13 | Smithburn 1949 [2] |
| Sore throat | 43 | 2 | 5 | Swanepoel 1979 [5] |
| **Gastro-enteritis syndrome** | | | | |
| Coated tongue | 8 | 2 | 25 | Smithburn 1949 [2] |
| Dysphagia | 43 | 2 | 5 | Swanepoel 1979 [5] |
| Odynophagia | 31 | 1 | 3 | Boushab 2016 [6] |
| **Hepatic syndrome** | | | | |
| Ascites | 165 | 4 | 2.4 | Mohammed A 2003 [4] |
| **Neurological syndrome** | | | | |
| Drowsiness | 13 | 12 | 92 | Abdel-Wahab 1978 [7] |
| Hypersalivation | 17 | 3 | 18 | Van Velden 1977 [8] |
| Neck pain | 8 | 1 | 13 | Smithburn 1949 [2] |
| Lack of gustatory discrimination | 8 | 1 | 13 | Smithburn 1949 [2] |
| Asthenia | 17 | 2 | 12 | Faye 2007 [9] |
| Visual hallucinations | 17 | 1 | 6 | Van Velden 1977 [8] |
| Irritability | 194 | 10 | 5 | El Imam 2009 [10] |
| Decelebrate posturing | 22 | 1 | 5 | Laughlin 1979 [11] |
| Neck stiffness | 48 | 2 | 4.1 | Kahiry 2005 [12] |
| Tremors | 475 | 7 | 1.5 | Madani 2003 [13] |
| Amnesia | 475 | 6 | 1.3 | Madani 2003 [13] |
| **Haemorrhagic syndrome** | | | | |
| Hypotension | 3 | 1 | 33.3 | Kitchen 1933 [14] |
| Haemorrhagic meningo-encephalitis | 31 | 4 | 31 | Boushab 2016 [6] |
| Disseminated intravascular coagulopathy | 165 | 31 | 19 | Mohammed A 2003 [4] |
| Hematuria | 194 | 20 | 10 | El Imam 2009 [10] |
| Rectal bleeding | 194 | 14 | 7 | El Imam 2009 [10] |
| Bleeding from puncture sites | 494 | 12 | 2.4 | Madani 2003 [13] |
| **Visual syndrome** | | | | |
| Red eye | 48 | 21 | 43.7 | Kahiry 2005 [12] |
| Retinal haemorrhage | 22 | 1 | 5 | Laughlin 1979 [11] |
| Complete blindness | 683 | 8 | 1.2 | Madani 2003 [13] |
| **Obstetric syndrome** | | | | |
| Abortion / miscarriage | 28 | 15 | 54 | Baudin 2016 [15] |
| Pre-term delivery | 28 | 1 | 3 | Baudin 2016 [15] |
| Normal pregnancy | 28 | 12 | 43 | Baudin 2016 [15] |
| **Cardio-pulmonary syndrome** | | | | |
| Chest pain | 8 | 1 | 13 | Smithburn 1949 [2] |
| Dyspnoea | 31 | 3 | 10 | Boushab 2016 [6] |
| Hiccups | 31 | 2 | 6 | Boushab 2016 [6] |
| Myocarditis | 43 | 2 | 5 | Swanepoel 1979 [5] |
| Pneumonia | 43 | 1 | 2 | Swanepoel 1979 [5] |
| **Laboratory manifestations** | | | | |
| Elevated gamma glutamyl transferase (GGT) | 86 | 23 | 26.7 | Mohammed A 2003 [4] |
| Elevated creatine phosphokinase (CPK) | 155 | 50 | 32.3% | Mohammed A 2003 [4] |

**References**

1. Kahlon SSP, C. J.; LeDuc, J.; Muchiri, E. M.; Muiruri, S.; Njenga, M. K.; Breiman, R. F.; White Jr, A. C.; King, C. H. Case report: Severe rift valley fever may present with a characteristic clinical syndrome. American Journal of Tropical Medicine and Hygiene. 2010;82(3):371-5. doi: <http://dx.doi.org/10.4269/ajtmh.2010.09-0669>. PubMed PMID: 358507949.
2. Smithburn K, Mahaffy A, Haddow A, Kitchen S, Smith J. Rift Valley fever: accidental infections among laboratory workers. The Journal of Immunology. 1949;62(2):213-27.
3. Francis T, Magill T. Rift Valley fever: a report of three cases of laboratory infection and the experimental transmission of the disease to ferrets. Journal of Experimental Medicine. 1935;62(3):433-48.
4. Al-Hazmi M, Ayoola EA, Abdurahman M, Banzal S, Ashraf J, El-Bushra A, et al. Epidemic Rift Valley fever in Saudi Arabia: a clinical study of severe illness in humans. Clinical infectious diseases. 2003;36(3):245-52.
5. Swanepoel RM, B.; Watt, J. A. Fatal Rift Valley fever of man in Rhodesia. Central African Journal of Medicine. 1979;25(1):1-8. PubMed PMID: 421262.
6. Boushab BM, Fall-Malick FZ, Ould Baba SEW, Ould Salem ML, Belizaire MRD, Ledib H, et al. Severe Human Illness Caused by Rift Valley Fever Virus in Mauritania, 2015. Open forum infectious diseases. 2016;3(4):ofw200-ofw. doi: 10.1093/ofid/ofw200. PubMed PMID: 27844026.
7. Abdel-Wahab KSEDEB, L. M.; El-Tayeb, E. M.; Omar, H.; Ossman, M. A. M.; Yasin, W. Rift Valley Fever virus infections in Egypt: pathological and virological findings in man. Transactions of the Royal Society of Tropical Medicine and Hygiene. 1978;72(4):392-6.
8. Van Velden DJJM, J. D.; Olivier, J. Rift Valley fever affecting humans in South Africa. A clinicopathological study. South African Medical Journal. 1977;51(24):867-71. PubMed PMID: 8135309.
9. Faye OD, M.; Diop, D.; Bezeid, O. E.; Ba, H.; Niang, M.; Dia, I.; Mohamed, S. A. O.; Ndiaye, K.; Diallo, D.; Ly, P. O.; Diallo, B.; Nabeth, P.; Simon, F.; Lo, B.; Diop, O. M. Rift valley fever outbreak with East-Central African virus lineage in Mauritania, 2003. Emerging Infectious Diseases. 2007;13(7):1016-23.
10. El Imam MES, M.; Omran, M.; Abdalkareem, A.; El Gaili Mohamed, M. A.; Elbashir, A.; Khalafala, O. Acute renal failure associated with the Rift Valley fever: a single center study. Saudi Journal of Kidney Diseases & Transplantation. 2009;20(6):1047-52. PubMed PMID: 19861868.
11. Laughlin LWM, J. M.; Strausbaugh, L. J.; Morens, D. M.; Watten, R. H. Epidemic Rift Valley fever in Egypt: observations of the spectrum of human illness. Transactions of the Royal Society of Tropical Medicine and Hygiene. 1979;73(6):630-3.
12. Kahiry W. Pattern of positive Rift Valley Fever (RVF) cases during the epidemic period Sep.-Dec. 2000 in Al-Zuhrah District-Hodiedah Governorate- Yemen. University of Aden Journal of Natural and Applied Sciences. 2005;9(3):597-607.
13. Madani TA, Al-Mazrou YY, Al-Jeffri MH, Mishkhas AA, Al-Rabeah AM, Turkistani AM, et al. Rift Valley fever epidemic in Saudi Arabia: epidemiological, clinical, and laboratory characteristics. Clinical Infectious Diseases. 2003;37(8):1084-92.
14. Kitchen SF. Laboratory infections with the virus of Rift Valley fever. 1934.
15. Baudin M, Jumaa AM, Jomma HJ, Karsany MS, Bucht G, Näslund J, et al. Association of Rift Valley fever virus infection with miscarriage in Sudanese women: a cross-sectional study. The Lancet Global Health. 2016;4(11):e864-e71.
