# Supplementary material for: Clinical manifestations of Rift Valley fever in humans: Systematic review and meta-analysis
Source: PLoS Negl Trop Dis. 2022 Mar 25;16(3):e0010233. doi: 10.1371/journal.pntd.0010233 (PMC8986116; doi:10.1371/journal.pntd.0010233)
Supplement: Attachment — Submitted filename: Response to reviewers comments.docx [file pntd.0010233.s022.docx]

**Clinical manifestations of Rift Valley fever in humans: Systematic review and meta-analysis" (PNTD-D-21-00768)**

*Dear Anita K. McElroy, MD, PhD
Associate Editor
PLOS Neglected Tropical Diseases

Dear A. Desiree LaBeaud
Deputy Editor
PLOS Neglected Tropical Diseases*

*Thank you for the detailed and careful review of our manuscript and sharing the feedback from the reviewers. Below is our point-by-point response to the comments raised by the reviewers.*

***Point-by-point response to the reviewers’ comments***

**Reviewer #1:** Methods and statistical analyses are appropriate to the scope of the study.

***Authors response***

*Thank you for the acknowledgement.*

**Reviewer #2:** • Table 2 requires clarification of using the term “In- and Out-patients” separately from the “In-patients” or “Out-patients”. Please explain the rationale in text.

***Authors response***

*Clarification has been added in the legend (line 206 - 209) as well as in the text (lines 150 - 156) as follows:*

*“For “In-patients”, the subjects source in the study were solely hospital based patients requiring admission; “Out-patients”, the subjects source in the study were only hospital based patients requiring no admission; “In-patients and out-patients”, the subjects source in the study were both hospital based patients requiring admission and patients requiring no admission and data collection in the included studies was combined; whereas “Community patients” referred to subjects whose source in the study was non-hospital based patients found in the community or at home. The rationale for this stratification by the reviewers was to reflect the level of severity of disease among patients included in the studies.”*

**Reviewer #3:** The objectives of the study were clearly stated in the author summary and introduction of the manuscript. The authors state that the study is to conduct a “systematic review and meta-analysis of existing literature aimed at determining the frequency and scope of clinical and laboratory manifestations of RVF in humans”. In addition, the authors highlighted and outlined 5 main learning points for their analysis.
The study design was a systematic review of literature and was done using standardized protocols and was appropriate given the stated objectives. The methodology and revie3w of manuscripts to include in the systematic review was also appropriate and in total 32 manuscripts were used for data extraction and analysis. Although this seems to be a small number it is likely there is not as an extensive body of literature containing the specific requirements laid out by the authors for data to include. And as the authors state in the discussion, there are likely more non-English papers containing relevant data but were not included in this analysis.
No ethical concerns were noted.

***Authors response:***

*Thank you.*

**Results**

**Reviewer #1:** Results are presented clearly and adequately.

***Authors response***

*Thank you.*

**Reviewer #2:** • Figure legends are not found for all images.

***Authors response*** *Figure legends have been added for all images and tables.*

**Reviewer #3:** The analysis of the systematic review and clinical case data was appropriate and straight forward. All analysis and results were descriptive of the findings from the manuscripts reviewed. As the authors stated, most for the clinical manifestations described would likely be from patients with more severe or late stage RVF disease. Although this may bias the presentation of results it does seem relevant from a public health standpoint considering mild illness is non-fatal and resolves on its own, but the results analyzed will greatly underestimate the true number of RVF infections. The authors did not analyze or compare results or studies of serological evidence of RVF infection as a comparison to the acutely ill case studies to provide an estimate of what percent of the clinically relevant cases presented in this manuscript to the total estimated RVF infections in the population.

The tables and figures accurately represent the data presented and are thorough and detailed.

***Authors response***

*Thanks so much for this significant observation. True we did not estimate the proportion of clinically relevant acute cases presented in this manuscript to the total estimated acute RVF infections in the population. We were able to establish the number of clinically relevant cases in each included manuscript (which would form the numerator), however, information was not available on the total number of people that were positive for RVF in the general population (which would form the denominator). Most studies enrolled patients that presented to hospital with signs and symptoms of RVF from whom screening was done and those confirmed to have RVF by a laboratory test enrolled. Thus we have added this point to our discussion in line 410 -415 as follows:*

*“In this study, we did not analyze results from studies of serological evidence of acute RVF infection so as to provide an estimate of the percentage of clinically relevant cases to the total estimated RVF infections in the population. Included studies reported on the number of acutely ill or symptomatic cases (which would form the numerator) but not on the total number of people that were positive for RVF in the general population (which would form the denominator). Serosurveys during RVF outbreaks would be a valuable adjunct to clinical case focussed studies.”*

**Conclusions**

**Reviewer #1:** Conclusions summarized in “Key learning points” require attention as indicated in the Summary and General Comments of the review.

***Authors response***

*All the key learning points have been revised and are now more aligned to the results of our review (lines 60 - 70) in response to the comments provided by the reviewer under “Summary and General Comments”.*

**Reviewer #2:** Clearly described the significance of this metanalysis.

***Authors response***

*Thank you.*

**Reviewer #3:** The overall discussion and conclusions stated by the authors agrees with the data presented. In general the authors discuss the complexity and broad range of clinical manifestations and signs and symptoms associated with RVF infection. The authors also explain that the RVF clinical picture can be associated with many other more common tropical infections and may not always be recognized at first as suspect RVF when cases initially seek medical care.

***Authors response***

*This is true. Thank you.*

**Reviewer #3:** The authors do recognize this analysis does have some limitations, They state that most of the manuscripts included in the analysis would be considered the more severe end of the RVF clinical spectrum, thus biasing the analysis and clinical data associated with RVF infection. They also state that most studies used in the analysis are small sample size and limited in detailed clinical data. In addition, the authors mention that this was not a meta analysis and limiting identification of symptoms more common to RVF.

***Authors response***

*The statement “…. the authors mention that this was not a meta analysis …” is not correct. We did consistently state throughout the manuscript that this was a systematic review and metanalysis of the frequency of RVF clinical manifestations in humans.*

**Reviewer #3:** The authors spend much of the discussion detailing the biological, immunological or virological explanations for the clinical manifestations identified in their analysis. Although this does add some good context and background for these clinical manifestations, I did not think the authors focused enough on the real-world relevance of their findings. The authors mention that this analysis would help improve routine surveillance, case finding and “one-health” policies. There was no detailed discussion related to these points. In fact the authors state that proposing a case definition form the analysis would be “reductionistic”. I think the authors could have expanded on the public health and surveillance relevance of their findings to how to improve and identify the most common clinical manifestations of RVF and helped propose which clinical manifestations would be best to prioritize suspect RVF cases.

***Authors response***

*Thanks for the detailed study of our work. We have made a keen reflection on our earlier rationale for not specifying a case definition and thus developed a clinical case definition for RVF (lines 301 -308). In effect, this has led to significant changes in our discussion.*

*Our discussion has expounded on the real world relevance of our findings by showing the link between our work and clinical case detection by health care workers, outbreak investigations, disease surveillance teams and how this can be enhanced by epidemiological RVF predictive information from other ‘one-health’ players in the veterinary and environmental sectors.*

*Our proposed RVF clinical case definition is:*

*A suspected RVF case can be clinically defined as any human subject presenting with the general febrile/flu-like and hepatic syndromes whether or not associated with the gastrointestinal, renal, neurological, haemorrhagic, obstetric, visual, and cardio-pulmonary syndromes and death. The detailed manifestations in patients would be as shown in the table below.*

***Patients classically present***

1. *General febrile/flu-like syndrome*

*Common: fever, headache, arthralgia, myalgia, chills, malaise, backache, fatigue, lethargy, rash.*

*Rare: weight loss, prostration, nasal congestion, dehydration, joint stiffness, sore throat*

2. *Hepatic syndrome*

*Common: elevated ALT, elevated AST, elevated LDH, jaundice, right hypochondriac tenderness, hepatomegaly, liver failure, splenomegaly*

*Rare: ascites, elevated gamma glutamyl transferase (GGT)*

***And/or any of the following forms***

3. *Gastrointestinal syndrome*

*Common: epigastric discomfort, vomiting, nausea, anorexia, abdominal pain, diarrhoea, constipation.*

*Rare: coated tongue, dysphagia, odynophagia*

4. *Renal syndrome*

*Common: renal failure, elevated creatinine*

*Rare: elevated creatine phosphokinase (CPK)*

5. *Neurological syndrome*

*Common: dizziness, delirium, insomnia, CNS symptoms/encephalitis, hyperaesthesia, coma, vertigo, meningismus, confusion, disorientation, hallucinations, ataxia, choreiform movements, hemiparesis, locked-in-syndrome*

*Rare: drowsiness, hypersalivation, neck pain, lack of gustatory discrimination, asthenia, visual hallucinations, irritability, decerebrate posturing, neck stiffness, tremors, amnesia*

6. *Haemorrhagic syndrome*

*Common: pallor, haemorrhagic disease, epistaxis, haematemesis, melena, shock, bleeding gums, sub-conjunctival haemorrhage, petechiae, haematochezia, uterovaginal bleeding, ecchymoses, macular/purpura rash, haemoptysis, thrombocytopenia, anaemia (low Hb)*

*Rare: hypotension, haemorrhagic meningo-encephalitis, disseminated intravascular coagulation, haematuria, rectal bleeding, bleeding from puncture sites*

7. *Visual syndrome*

*Common: injected conjunctiva, eye pain, retro-orbital pain, blurred or partial blindness, photophobia, retinitis*

*Rare: red eye, retinal haemorrhage, complete blindness*

8. *Obstetric syndrome*

*Rare: abortion / miscarriage, pre-term delivery*

9. *Cardio-pulmonary syndrome*

*Common: syncope, throat swelling, cough*

*Rare: chest pain, dyspnoea, hiccup, myocarditis, pneumonia*

10. *Death - common*

*Legend: Common – two or more studies reported on the sign/symptom and a pooled prevalence estimate could be calculated using random effects meta-analysis; Rare – a single study reported on the sign/symptom and a pooled prevalence estimate could not be calculated using random effects meta-analysis.*

*We have indicated in the discussion that this case definition needs to be used in concurrence with other epidemiological factors, such as mass abortions or confirmation of RVF in animals, and that RVF can only be definitively diagnosed in the laboratory.*

**Reviewer #3:** The authors also listed 5 “Key Learning Points” at the beginning of their manuscript.

1. Human RVF outbreaks can occur any time of the year in endemic areas and over the years there has been an increased reporting of the disease among women.
2. RVF presents with diverse clinical manifestation which are inherently related to the nature of the infecting virus strains hence human disease is best defined inform of clinical syndromes.
3. A clear and detailed definition of human disease is likely to improve case detection during routine surveillance and outbreaks, estimation of disease burden, and consequently inform “one-health” policies in endemic countries.
4. Gaps exist on the link between RVF and human obstetric outcomes, the ability to induce autoimmunity, and its persistence in body tissues beyond what is currently known.
5. Airborne transmission is possible thus underlines the need for development and licensure of RVF vaccines and antivirals.
The authors do address all of these points in their paper, but I would question inclusion of some based on the low priority in which they were discussed in the manuscript. The authors provided detailed explanation of points #2 and #4 and to a lesser extent #1. I feel points # 3 and #5 were not sufficiently supported.

***Authors response***

*All the key learning points have been revised and new ones more aligned to the results of our review have been developed (lines 60 - 70) in response to the comments provided by the reviewer under “Summary and General Comments. Each key learning point has been expounded in the discussion. The new key learning points are:*

1. *Human RVF virus disease present in the form of nine clinical syndromes including the general febrile or flu-like, hepatic, gastrointestinal, neurological, haemorrhagic, visual, renal, cardio-pulmonary and obstetric syndromes; and death.*
2. *The human clinical picture is non-specific and overlaps in similarity to that of other endemic infections and patients can be co-infected by these pathogens further complicating RVF disease presentation.*
3. *Less than half of the studies used a case definition and a clear and detailed clinical case definition of human disease was lacking.*
4. *Most patients with the severe forms of disease require hospitalisation and commonly die from hepatic and/or renal failure and shock.*
5. *There was increased reporting of disease among women over the years yet studies are lacking on the association between RVF and human miscarriages and congenital anomalies.*

**Reviewer #3:** A stated above, the authors did not propose or discuss a detailed case definition or how one based on their findings would improve surveillance or one health policies. They in fact stated the opposite and suggested the clinical manifestations were too complex and broad to do so and a syndromic approach would be best.

***Authors response***

*A case definition for RVF has been proposed (see lines 301 -308) and the discussion has been improved to indicate how one based on syndromic grouping is beneficial to the frontline health care workers, epidemiologists investigating outbreaks and surveillance teams. We have also indicated how if the syndromic surveillance approach is embraced by the human ministry as a surveillance system for RVF can be enhanced by alerts of increased RVF activity signals from the veterinary and environmental/climatological surveillance systems.*

**Reviewer #3:** For point #5, the only mention of this in the discussion was a single case from Egypt. This could be argued, if in fact true, is a very rare occurrence and could have been caused by a number of clinical procedures causing aerosolized RVF to infect a physician. This does not seem to be a primary learning point and not a major finding of this analysis.

***Authors response***

*This has been removed from the key learning points. True, there are no studies about human-human airborne transmission, however, there is a genuine risk associated with air borne transmission. Other authors as cited in the discussion have reported on the risks associated with aerosol inhalation by herders handling infected tissues or assisting animal births, abattoir workers involved in animal slaughtering and dressing, veterinarians conducting autopsies as well as previous accidental laboratory infections. We believe this risk shouldn’t be negated as there are no large scale studies of acute RVF infections in humans. We have reinforced this point in the discussion by adding more text on these potential sources of aerosol transmission (lines 417 - 421).*

**Editorial and Data Presentation Modifications**

**Reviewer #1**: No editorial suggestions.

***Authors response***

*Thank you.*

**Reviewer #2:** • Line 91: “In the year 2000, a major outbreak occurred for the first time in the Arabian Peninsula [5, 6], and human cases have been reported in non-endemic countries such as the United States of America (USA) [7, 8], United Kingdom (UK) [9], and China [10]”. This sentence should be separated into two, because those listed RVF cases in the U.S. reported in 1930s.

***Authors response***

*Thanks for the suggestion. The sentence has been separated into two. More information has been added for clarity to the US/UK cases to specifically indicate they were “accidental laboratory infections”, unlike the community outbreaks of the Arabian Peninsula.*

*The section has been re-written as follows (lines 105 – 108.): “In the year 2000, a major outbreak occurred for the first time in the Arabian Peninsula [**5**,* *6**], and in 2016 one case of RVF initially thought to be yellow fever was imported into China [**7**]. Human cases of accidental laboratory infections with RVF have been reported in non-endemic countries such as the United States of America (USA) [**8**,* *9**] and United Kingdom (UK) [**10**].”*

**Reviewer #2:** • Line 329: “The NSs protein also forms filamentous structures that interact with nuclear chromatin to cause chromosomal segregation and defects [59, 60] and this is believed to be the mechanism for the congenital anomalies and abortion storms in animals” Although the NSs proteins are considered virulence factor for RVF, the direct association with the abortion has not been demonstrated. Rather, Oymans J. et al. (PLoS NTD, 2020, 14: e0007898) demonstrated that RVFV infection in trophoblasts can trigger inflammation and hemorrhage in the placenta in sheep, which is likely the cause of abortion.

***Authors response***

*Thanks for the detailed review. The sentence has been corrected (lines 350 - 352) to read, “The NSs protein also forms filamentous structures that interact with nuclear chromatin to cause chromosomal segregation and cohesion defects [**59**,* *60**] and this is inferred to be partly the mechanism for the congenital anomalies and abortion storms in animals [**61**]”*

*The words “inferred” to be “partly” the mechanism, have been added to indicate that the interaction between the NSs proteins and nuclear chromatin and the link to congenital anomalies and abortion storms is not direct but was postulated by the authors of the papers referenced. This now clearly reflects what the authors meant.*

*In addition, we have supported the link between RVF virus infection and abortions by a recent publication by Oymans et al 2020 (lines 352 - 355) as follows: “In fact, a recent study by Oymans and colleagues found that the RVF virus directly invades sheep placental epithelial cells and fetal trophoblasts causing placental tissue necrosis, haemorrhage and subsequently induction of abortion. The virus was also found to replicate efficiently in human placental explants”.*

**Reviewer #2:** • Line 362: “In an RVF rhesus macaque model, the kidneys exhibited the highest concentration of the virus, implying it is a major site of viral replication [76].” This study showed RVFV titers in kidneys lower than those in livers or spleens, in which authors stated that “Titration of organ homogenates clearly established liver and spleen as major sites of viral replication, with kidney and lung also suspect”. Accordingly, authors should correct the interpretation.

***Authors response***

*Thanks for the keen observation. This was a slip-up in writing. We meant to say ‘liver’ and not ‘kidneys’. We absolutely agree, the highest titres were seen in the liver, followed by spleen, then kidneys. Therefore, this sentence has been corrected (line 390) to read, “In an RVF rhesus macaque model, the liver exhibited the highest concentration of the virus, implying it is a major site of viral replication [76].”*

**Reviewer #2:** • The RVF patient returned to China was initially misdiagnosed as Yellow Fever. It might be important to indicate how to distinguish RVF from other similar febrile illnesses.

***Authors response***

*We have indicated that it is clinically difficult to differentiate RVF from other endemic tropical infections such as yellow fever, hepatitis, chikungunya, malaria, dengue fever, etcetera (lines 470 - 483). We have indicated that it is important to link the clinical suspicion to the epidemiological abortion storms and new born deaths in livestock, although this may not be so in case the patient is a traveller, had an accidental laboratory infection or the pathogen is used as a bioterrorism agent targeting humans. And that the distinction from these infections should be made definitively through the laboratory.*

**Reviewer #3:** none

***Authors response***

*Thank you.*


**Summary and General Comments**

**Reviewer #1:** The review by Anywaine et al. on clinical manifestations of RVF in humans based on systematic review and meta-analysis is a useful contribution that outlines the complex symptomatology of human RVF disease into syndromes. This approach might assist clinicians and epidemiologist in defining the disease case definition, thus has a potential to improve RVF detection rates, syndromic disease surveillance, outbreaks control, and increase public awareness.
The authors should consider not overstating frequency of severe symptoms in RVFV-infected humans.

***Authors response***

*We have indicated that most patients present with the general febrile syndrome and elevation in liver transaminases which are a mild form of the disease. We have also indicated among the limitations of our work that “most studies enrolled mostly inpatients and outpatients (rather than patients from the community) which biases the overall picture to a more severe disease”.*

**Reviewer #1:** Description of RVF as a “mosquito-borne viral haemorrhagic zoonosis associated with severe morbidity” undermines the common clinical course of RVFV infection in humans, which is mostly moderate self-limiting febrile disease.

***Authors response***

*This has been corrected. The sentence has been toned down by deleting the words “haemorrhagic” and “severe”. The sentence now reads (line lines 18, 19), “Rift Valley fever (RVF) is an emerging, neglected, mosquito-borne viral zoonosis associated with significant morbidity, mortality and expanding geographical scope.”*

**Reviewer #1:** More specific comments to be addressed:
“Human symptoms have poor specificity” it should be rather “Clinical manifestations (signs and symptoms) in human are unspecific”

***Authors response***

*This has been corrected (lines 19, 20) to read “The clinical signs and symptoms in human are non-specific and case definitions vary”.*

**Reviewer #1:** Authors claim that they “determined the clinical manifestations of RVF in humans” where in fact they review and analyzed them.

***Authors response***

*This has been corrected (lines 20, 21) to read “We reviewed and analysed the clinical manifestations of RVF in humans”*

**Reviewer #1:** “Death occurred in 21% (95% CI 14-29; [16 studies, 328 patients]) of cases. These results should be more carefully or more responsibly interpreted because they imply relatively very high case fatality rate among RVF-infected people. It should explained that these results mostly concern hospitalized cases. Most community infections with RVFV are asymptomatic or mild.

***Authors response***

*Slightly more description has been added to the sentence in the abstract (lines 35, 36) to read, “Death occurred in 21% (95% CI 14-29; [16 studies, 328 patients]) of cases, most of whom were hospitalised.” In the results section (lines 297, 298), clarity has been added as “Death occurred in 21% (95% CI 14-29; [16, 328]) of cases, mostly among those that sought care from the hospital.”*

*We have added a paragraph to the discussion on the patient categories that die and the causes of death. The section in the discussion (lines 396 - 410) is as follows: “In this review one-fifth (21%) of patients with RVF clinical signs and symptoms succumbed to death and these were mostly those who attended the hospital as in-patients and outpatients. A similar picture of disease severity was reflected across all the other syndromes presented in table 2. Most of the patients reported in the included studies had severe forms of disease (neurological, hepatic, renal, haemorrhagic and visual syndromes). This is possibly not a representative picture of the disease in the community as severely sick patients are more likely to seek care than those with mild symptoms and hospitalised severe patients more likely to deteriorate to death. In this review, the commonest causes of death include isolated acute hepatic or renal failure, compound hepatorenal impairment, and shock within the first week of illness [**30**,* *31**,* *40**,* *41**]. Much as this mortality is biased towards those with severe disease, human mortality from RVF has been observed to increase over the years. A systematic review conducted in 2015 by Nanyingi et al, reported human mortality during outbreaks ranging from 0.3% to 44.7% [**4**]. Whether this is related to increasing viral virulence or different biases in case finding and reporting is not known [**83**], however, it is important to intensify supportive patient care to prevent or avert liver, renal and cardiovascular failure.”*

**Reviewer #1:** In “Author summary: the authors stated that “limited surveillance and estimation of disease burden ….is due to the inability to concisely define the disease” This is too simplified. One of the major problem in RVF-endemic countries with diagnosis of RVF (notably Africa) is limited diagnostic capacity, lack of well-structured and long-term surveillance studies, including inter-epidemic periods and long inter-epidemic periods that make justification for such as studies difficult.

***Authors response***

*In the author summary this section (lines 48, 49) has been corrected to read “…there is limited surveillance and estimation of disease burden which in part is due to the inability to concisely define the disease”.*

**Reviewer #1:** The “Key learning points” require most attention.

***Authors response***

*Thanks so much for highlighting the issues with our key learning points. All the key learning points have been revised and now formulated in line with the results of the study.*

**Reviewer #1:** “Human RVF outbreaks can occur any time of the year in endemic areas”
Is this true? Are RVFV vectors active all the year around in all RVF-endemic regions?

***Authors response***

*This point was removed from among the key learning points. However, the sentence has been refined in the discussion section (lines 462 - 465.) for more clarity that; “This review showed that human RVF outbreaks have occurred in different countries at different time points in a year, though mostly in March/April and October/November. This observation aligns well with the rainy seasons in East Africa which are known to peak in the same months with occasional intraseasonal and interannual variability in different countries.*

**Reviewer #1:** “RVF presents with diverse clinical manifestation which are inherently related to the nature of the infecting virus strains”. This statement suggest that diversity of clinical manifestations depend on specific nature/ability of a RVFV strain involved or that different RVFV strains are responsible for different clinical outcomes in humans. Is this supported by any study in humans?

***Authors response***

*This has been removed as a key learning point.*

*In our discussion lines 330 – 341, we indicated that the nature of RVF virus strain is not the only factor responsible for the diverse clinical manifestations of RVF but both viral and host factors are responsible. Among the viral factors, viral tissue tropism could be one of the factors associated with clinical diversity. We have however made some clarification with the right references that RVF in its natural form is pantropic although the hepatotropic form predominates in most outbreaks. And that one accidental laboratory infection with the neurotropic form had a hepatotropic virus recovered instead. We have indicated that natural existence of neurotropic forms in humans has not been demonstrated and that whilst no human studies have been conducted to conclusively study the association between RVF tropic nature and its symptomatology, it is possible this multifarious nature could partly be responsible for the dominance of certain syndromes in some outbreaks.*

*We did also indicate that virulence could result from genetic re-assortment and cited a study by Liu et al 2017 in which a patient who developed a cytokine storm and severe clinical disease had the S-segment re-assorted from the A viral lineage which is known to be highly pathogenic and caused neary 600 deaths in the 1977/8 outbreak in Egypt.*

**Reviewer #1:** “A clear and detailed definition of human disease is likely to improve case detection during routine surveillance and outbreaks, estimation of disease burden, and consequently inform “one-health” policies”. The later conclusion on how it will inform “One Health” policies is unclear and should be explain.

***Authors response***

*This has been removed as a key learning point.*

*Clarity has been added to the discussion. We developed a clinical case definition which is based on the syndromic categorisation of manifestations. We have indicated that syndromic surveillance for human RVF could be enhanced by alerts of increased RVF virus activity from the veterinary and environmental sector predictive tools. These inter-sectoral collaborations could enhance the ‘one-health’ disease control strategies.*

**Reviewer #1:** “Gaps exist on the link between RVF and human obstetric outcomes, the ability to induce autoimmunity, and its persistence in body tissues beyond what is currently known”.
The review is not supporting or is not giving sufficient background/facts to justify that there are knowledge gaps and thus a need for studies on RVFV ability to induce autoimmunity disease or to establish a persistence /chronic state in body tissues.

***Authors response***

*This key learning point has been re-phrased (lines 69, 70) as follows, “There was increased reporting of disease among women over the years yet studies are lacking on the association between RVF and human miscarriages and congenital anomalies.”*

*Correction has been made in the discussion (line 382-383) by deleting the statement “Further research is needed on the role of RVF viruses in inducing autoimmunity in humans and other susceptible animal species”.*

*The suggestion about further research to establish viral persistence has also been removed. The statement “Another hypothesis is” has been deleted.*

*The entire section (lines 381 - 385) now reads “This autoimmune mechanism could presumably be responsible for other late manifestations in other tissues. On the other hand, it could be that the magnitude and avidity of antibodies produced is variable (i.e. low and ineffective) such that the virus persists in some body tissues longer than is currently known causing more injury.”*

**Reviewer #1:** “Airborne transmission is possible thus underlines the need for development and licensure of RVF vaccines and antivirals”
Again, authors overemphasize the RVF as a formidable public health threat. This is not necessary to justify its public health importance. Also, note that RVF epidemic-prone potential is not driven by airborne transmission, thus “airborne transmission” is misused to justify the need for the development of vaccines and antivirals.

***Authors response***

*This has been removed as a key learning point. However, there is a genuine risk associated with air borne transmission. Other authors as cited in the discussion (lines 417 - 421) have reported on the risks associated with aerosol inhalation by herders handling infected tissues or assisting animal births, abattoir workers involved in animal slaughtering and dressing, veterinarians conducting autopsies as well as previous accidental laboratory infections. We believe this risk shouldn’t be negated as there are no large scale studies of acute RVF infections in humans. We have reinforced this point in the discussion by adding more discussion on these potential sources of aerosol transmission.*

**Reviewer #2:** The manuscript entitled “Clinical manifestations of Rift Valley fever in humans: Systematic review and metaanalysis” by Anywaine Z., et al. analyzed selected 32 reports for the meta-analysis of common RVF clinical signs. This study was performed to improve the poor RVF case definition used to analyze past RVF outbreaks. Overall, this manuscript is well written and captured representative RVF symptoms with the estimated proportions. There are several points requiring further clarifications.

***Authors response***

*Thank you. All points documented by the reviewer have been revised and clarified.*

**Reviewer #3:** Overall, this manuscript is a good review of available English language manuscripts on RVF clinical manifestations of illness. It does consolidate a broad range of findings and summarizes them well. I suggest the discussion expand on the real-work and public health relevance of the findings more and revise the key learning points.

***Authors response***

*Thank you so much for the comments and suggestions. The key learning points have been revised and replaced with new ones aligned to the work.*

*We have developed a clinical case definition for RVF by condensing the diverse manifestations under syndromic groupings. Our discussion has elaborated on its applicability and the significance of syndromic approach to RVF routine clinical case diagnosis and surveillance. We have indicated how the syndromic surveillance can be enhanced by RVF predictive epidemiological statistics from the veterinary and environmental surveillance sectors.*
