# Supplementary material for: Clinical manifestations of Rift Valley fever in humans: Systematic review and meta-analysis
Source: PLoS Negl Trop Dis. 2022 Mar 25;16(3):e0010233. doi: 10.1371/journal.pntd.0010233 (PMC8986116; doi:10.1371/journal.pntd.0010233)
Supplement: Attachment — Submitted filename: PNTD-D-21-00768.docx [file pntd.0010233.s023.docx]

The authors did a systematic review and meta-analysis of the clinical manifestations of Rift Valley fever in humans and describe the complexity and variety of symptoms of Rift Valley fever in humans by grouping the symptoms into syndromes. The manuscript is well written and I just have a few comments and suggestions listed below.

Abstract

Page 1 line 21-23: why are the dates not the same – perhaps just mention why one is up to 13^th^ October, another up to 14^th^, and the last to up to the 15^th^

Page 2 line 28: suggest to replace “, 32” with “…less than 1% (32)...”

Page 2 line 31-34: suggest to rearrange the CI’s in decreasing order

Page 2 line 34: Death occurred in 53% in results – but 21% in abstract?

Introduction

Page 4 line 88: I would personally remove “emerging” and “haemorrhagic” – RVF has been around for quite some time and only sometimes cause haemorrhage – rather state why it is of concern for public health worldwide

Page 4 line 95: Not all countries have inadequate laboratory capacity – some African laboratories are world leaders when it comes to RVFV research – rather rephrase to state majority of labs does not have adequate lab capacity

Page 4 line 96: remove extra space before “burden”

Methods

Page 5 line 118: replace “excel” with “Microsoft Excel (Microsoft Corporation)”

Page 5 line 122 and 124: remove extra space after full stop

Page 5 line 134: suggest to replace “etcetera” with “…to mention a few.”

Page 6 line 150: replace “…in a table.” with “…in table form.”

Results

Page 10 line 179-180: I would like to see this broken down a little bit into climatic regions – temperate regions vs tropical vs arid – does these peaks co-inside with rainy seasons?

Page 13 line 209: replace “flu” with “influenza”

Page 14 line 236 and 241: keep consistent – inpatient care vs in-patient admission

Page 14 line 244-247: suggest to rearrange the numbers

in decreasing order

Discussion

Page 16 line 307: replace “flu” with “influenza”

Page 17 line 337: add virus after “Zika.”

Page 17 line 338-339: with which season does these months coincide? Tropical vs temperate

Page 18 line 359: add virus disease after “Ebola and Zika”

Page 19 line 382: it is important to highlight that the case definition and clinical syndromes overlap with other arboviruses

Page 19 line 386: highlight that this is a one-health approach and the strengths of different disciplines working together

References

Some references are incomplete:

8, 14, 16, 24, 63, 64, 65, 67

Figure 1

Why were the 4 non-English articles of the 50 excluded articles (under the eligibility tab) not removed during the previous 11 articles of other languages

Figure 2

Very low resolution – cannot read any text on the figure

Figure 3a-b

Very low resolution – unable to read any text – could not review
