# Supplementary material for: Clinical manifestations of Rift Valley fever in humans: Systematic review and meta-analysis
Source: PLoS Negl Trop Dis. 2022 Mar 25;16(3):e0010233. doi: 10.1371/journal.pntd.0010233 (PMC8986116; doi:10.1371/journal.pntd.0010233)
Supplement: Attachment — Submitted filename: Response to reviewers comments.docx [file pntd.0010233.s024.docx]

**Clinical manifestations of Rift Valley fever in humans: Systematic review and meta-analysis" (PNTD-D-21-00768)**

*Dear Anita K. McElroy, MD, PhD
Associate Editor
PLOS Neglected Tropical Diseases

Dear A. Desiree LaBeaud
Deputy Editor
PLOS Neglected Tropical Diseases*

*Thank you for reviewing our second version of the manuscript and the responses to the reviewers’ comments following the initial version of the paper submitted in May 2021. We sincerely acknowledge that the review process has been informative and enriching to our work. Here below is our detailed point-by-point response to the comments raised by the reviewers.*

***Point-by-point response to the reviewers’ comments***

**Methods**

**Reviewer #2:** Authors revised unclear points in Methods section according to reviewers' suggestions.

**Authors response**
*Thank you.*

**Reviewer #4:** The objectives of the study were clearly stated.

**Authors response**
*Thank you.*

**Results**

**Reviewer #2:** Authors added Figure legends appropriately.

**Authors response**
*Thank you.*

**Reviewer #4:** Results were clear - the figures were not of sufficient quality to review.

**Authors response**

*Thank you for the comment. We did note that this comment refers to an earlier (first) version of the manuscript to which reviewers 1, 2 and 3 gave comments. A similar observation on the inadequate quality of figures was made by other reviewers of the initial manuscript. All figures were updated in the second version of the manuscript submitted which reviewer #4 did not look at.*

**Conclusions**

**Reviewer #2:** The manuscript has been improved for clarity after the revision.

**Authors response**
*Thank you.*

**Reviewer #4:** Conclusion was adequate.

**Authors response**
*Thank you.*

**Editorial and Data Presentation Modifications**

**Reviewer #2:** Adequately addressed all points.

**Authors response**
*Thank you.*

**Reviewer #4:** Minor Revision.

**Authors response**
*Thank you so much. Our action and responses to the suggested changes are indicated in the revised manuscript and in this document respectively.*

**Summary and General Comments**

**Reviewer #2:** The revised manuscript by Anywaine Z. et al. was adequately addressed this reviewer's points.

**Authors response**
*Thank you so much for sparing the time to review our work.*

**Reviewer #4:** Please see uploaded document.

**Authors response**

*Thanks so much for the keen review and for refining our manuscript. We have copied and pasted the review comments from the uploaded document below, and against each comment/suggestion is our response highlighting the action undertaken in line with the comment.*

**Reviewer #4:** The authors did a systematic review and meta-analysis of the clinical manifestations of Rift Valley fever in humans and describe the complexity and variety of symptoms of Rift Valley fever in humans by grouping the symptoms into syndromes. The manuscript is well written and I just have a few comments and suggestions listed below.

**Authors response**

*Thank you for the compliment.*

**Abstract**

**Reviewer #4:** Page 1 line 21-23: why are the dates not the same – perhaps just mention why one is up to 13^th^ October, another up to 14^th^, and the last to up to the 15^th^

**Authors response**

*Thank you for the comment. A statement “on different dates” has been added to the sentence for clarity. The sentence now reads “In this systematic review and meta-analysis we searched on different dates, the Embase (from 1947 to 13^th^ October 2019), Medline (1946 to 14^th^ October 2019), Global Health (1910 to 15^th^ October 2019), and Web of Science (1970 to 15^th^ October 2019) databases.”*

**Reviewer #4:** Page 2 line 28: suggest to replace “, 32” with “…less than 1% (32) ...”

**Authors response**

*Thanks for the suggestion. The sentence has been modified to read, “Of the 3765 articles retrieved, less than 1% (32 articles) were included in the systematic review and meta-analysis”.*

**Reviewer #4:** Page 2 line 31-34: suggest to rearrange the CI’s in decreasing order

**Authors response**

*Thanks for the suggestion, however, we have arranged the prevalence estimates (which are the main outcomes) in decreasing order.*

**Reviewer #4:** Page 2 line 34: Death occurred in 53% in results – but 21% in abstract?

**Authors response**

*Thanks for the observation. The “53%” figure in the results section (page 12, line 187 – 188) did not refer to cases of death but rather to the proportion of studies that reported on death. To add more clarity to the sentence, “53%” has been deleted, and the sentence re-written as, “Death was reported in 16 out of the 32 included studies.”*

**Introduction**

**Reviewer #4**: Page 4 line 88: I would personally remove “emerging” and “haemorrhagic” – RVF has been around for quite some time and only sometimes cause haemorrhage – rather state why it is of concern for public health worldwide

**Authors response**

*The words “emerging” and “haemorrhagic” have been deleted. Similarly, the phrase “that humans acquire from infected domestic and wild ruminants” is deleted since it was a duplication for the word “zoonosis” appearing earlier in the same sentence. Instead the significance to public health has been added and the sentence now reads, “Rift Valley fever (RVF) is a neglected, mosquito-borne and direct contact viral zoonosis associated with significant morbidity, mortality and an expanding geographical scope”.*

**Reviewer #4:** Page 4 line 95: Not all countries have inadequate laboratory capacity – some African laboratories are world leaders when it comes to RVFV research – rather rephrase to state majority of labs does not have adequate lab capacity.

**Authors response**

*Thanks for the comment, we have amended the sentence to read: “Further, majority of laboratories in areas prone to RVF outbreaks have inadequate diagnostic capacity [5, 16, 17], contributing to an underestimation of the RVF disease burden [**18**].”*

**Reviewer #4:** Page 4 line 96: remove extra space before “burden”

**Authors response**

*The word “burden” in the introduction section appears only in line 100 and there is no extra space before it.*

**Methods**

**Reviewer #4:** Page 5 line 118: replace “excel” with “Microsoft Excel (Microsoft Corporation)”

**Authors response**

*Thank you for the observation. Correction has been made in all sections where the word “excel” was inappropriately used instead of “Microsoft Excel” or “Microsoft Excel spreadsheet”.*

**Reviewer #4:** Page 5 line 122 and 124: remove extra space after full stop

**Authors response**

*There was no extra space after a full stop in lines 122 and 124. Instead extra spaces after a full stop were in lines 129 and 130 and these have been deleted. In addition, the entire manuscript has been checked for extra spaces and all found were deleted.*

**Reviewer #4:** Page 5 line 134: suggest to replace “etcetera” with “…to mention a few.”

**Authors response**

*The word “etcetera” is used in line 146. The word has been deleted and the sentence rephrased as “In addition, data were captured on variables such as laboratory tests used to confirm the diagnosis of RVF, number or proportion that had co-infections and the type of co-infections.”*

**Reviewer #4:** Page 6 line 150: replace “…in a table.” with “…in table form.”

**Authors response**

*This comment refers to line 162 in the current version of the manuscript. Correction has been made and the sentence now reads, “Results from the meta-analysis were reported using forest plots for each symptom and further summarised in table form”.*

**Results**

**Reviewer #4:** Page 10 line 179-180: I would like to see this broken down a little bit into climatic regions – temperate regions vs tropical vs arid – does these peaks co-inside with rainy seasons?

**Authors response**

*Thank you for the suggestion. Unfortunately, we did not collect information on the relationship of countries to the climatic strata and this is heterogeneous for different countries. However, in the discussion section (page 23, lines 448-451) of the revised manuscript we did indicate that “This review showed that human RVF outbreaks have occurred in different countries at different time points in a year, though mostly in March/April and October/November. These results align well with the rainy seasons in East Africa which are known to peak in the same months with occasional intra-seasonal and inter-annual variability in different countries [**93**]”.*

**Reviewer #4:** Page 13 line 209: replace “flu” with “influenza”

**Authors response**

*This comment refers to page 15 line 212. The word “flu” has been replaced with “influenza”. In addition, this has consistently been corrected throughout the entire manuscript with the word “flu” where used in reference to the syndrome, replaced with “influenza”.*

**Reviewer #4:** Page 14 line 236 and 241: keep consistent – inpatient care vs in-patient admission

**Authors response**

*This comment refers to page 15 line 239. The word “inpatient” has been corrected to “in-patient”. In addition, this correction has been made throughout the entire manuscript for consistency.*

**Reviewer #4:** Page 14 line 244-247: suggest to rearrange the numbers in decreasing order

**Authors response**

*This comment refers to page 16 lines 247-250. In our style of writing we did consistently present the pooled proportions under each syndrome in a decreasing order. However, where the number of studies and sample size onto which the pooled proportion was calculated was quite large compared to the number of studies and sample sizes of other symptoms, we presented the symptom earlier than other symptoms despite its lower pooled proportion. In the particular instance highlighted here the results are as follows: “Neurological manifestations included encephalitis (21%, 11-33; [8, 173]), dizziness (46%, 1-95; [4, 10]), delirium (42%, 12-74; [4, 13]) and insomnia (33%, 0-79; [2, 2]). In addition, patients presented with hyperaesthesia (13%, 0-35; [2, 2]), vertigo (9%, 0-31; [3, 20]), meningismus (7%, 0-20; [5, 14]), confusion (5%, 1-11; [6, 57]), disorientation (5%, 3-8; [2, 32]), and coma (9%, 4-16; [7, 52]).”*

*Encephalitis is presented first because we believe it is more common as it was reported in 8 studies and 173 patients. The rest of the symptoms were reported by few studies and in a small number of patients and the rest of the symptoms are presented in a decreasing order of their pooled proportions.*

**Discussion**

**Reviewer #4:** Page 16 line 307: replace “flu” with “influenza”

**Authors response**

*The word “flu” has been replaced with “influenza” and the syndrome is consistently corrected in the entire manuscript where the word flu was used to “General febrile/influenza-like syndrome”*

**Reviewer #4:** Page 17 line 337: add virus after “Zika.”

**Authors response**

*This comment refers to page 20 line 354. The word virus has been added after Zika.*

**Reviewer #4:** Page 17 line 338-339: with which season does these months coincide? Tropical vs temperate

**Authors response**

*In the current version of the manuscript, this comment refers to page 23 lines 448-451. In the earlier version of the manuscript to which this comment refers, we had not elaborated on the point to show the seasons that coincide with the peak months. In the updated version we did indicate that “This review showed that human RVF outbreaks have occurred in different countries at different time points in a year, though mostly in March/April and October/November. These results align well with the rainy seasons in East Africa which are known to peak in the same months with occasional intra-seasonal and inter-annual variability in different countries [**93**]”. Therefore, the seasons align well with the African tropical climate.*

**Reviewer #4:** Page 18 line 359: add virus disease after “Ebola and Zika”

**Authors response**

*This comment refers to page 21 line 374 -375. The phrase “virus disease” has been added after “Ebola and Zika” and the sentence now reads, “This has been observed with other viral infections such as Ebola and Zika virus disease [**78**,* *79**]. ”*

**Reviewer #4:** Page 19 line 382: it is important to highlight that the case definition and clinical syndromes overlap with other arboviruses.

**Authors response**

*In the current version of the manuscript, this comment was addressed on page 24 lines 456-459 as follows: “Despite the proposed case definition, it is clinically difficult to differentiate RVF from other endemic tropical infections. In this systematic review and meta-analysis, we included only laboratory confirmed RVF cases but still no distinct manifestation stands out to be peculiar to RVF. The disease picture overlaps in similarity to that of yellow fever, viral hepatitis, chikungunya, malaria, dengue fever, typhoid, to mention but a few.”*

**Reviewer #4:** Page 19 line 386: highlight that this is a one-health approach and the strengths of different disciplines working together

**Authors response**

*Thanks for the comment. In the revised version of the manuscript, this point was comprehensively discussed on page 23 lines 440-455. We did mention that “In RVF endemic countries it would be ideal for human surveillance programmes to embrace the syndromic approach to diagnosis or data generation and analysis so as to inform quick public health responses [**37**]. Signals for vigilance could be heightened by notifications of increased RVF virus activity from the veterinary and environmental sector surveillance programmes. Sentinel herds monitoring (SHM) of RVF virus activity is a common practice in the animal health sector. Similarly, climatologists use remote sensing satellite data (RSSD)….”. That, “These epidemiological predictive tools can be used to alert the human ministry to intensify on RVF surveillance ahead of any potential outbreak [**83**]. Practically this could enhance the currently loose ‘One-health’ inter-sectoral collaboration [**19**].”*

**References**

**Reviewer #4**: Some references are incomplete: 8, 14, 16, 24, 63, 64, 65, 67

**Authors response**

*All references have been updated and are now complete. Reference 8 is now 9, 14 is currently 15 and 16 was removed. Reference 24 is now 25, 63 is currently 68 (no update was required), 64 is 69, 65 is 70 and 67 is 72 (which was also complete). Other references completed include 47 and 48.*

**Figures**

**Reviewer #4:** Figure 1

Why were the 4 non-English articles of the 50 excluded articles (under the eligibility tab) not removed during the previous 11 articles of other languages

**Authors response**

*The subheading for the 11 articles is “11 articles not accessible excluded”. The full text versions of these papers could not be retrieved or could not be found and when we analysed them, 9 were French, 1 Spanish and 1 Dutch. This was a step before we applied the eligibility criteria. For the 4 non-English articles, we were able to retrieve them, applied eligibility criteria and excluded on the basis of language in which they were written.*

**Reviewer #4**: Figure 2

Very low resolution – cannot read any text on the figure

**Authors response**

*A new figure 2 has been uploaded.*

**Reviewer #4:** Figure 3a-b

Very low resolution – unable to read any text – could not review

**Authors response**

*This was observed by other reviewers of the initial manuscript version. These were updated in the current version of the manuscript which the reviewer did not look at.*
